# Integrated Metabolic and Inflammatory Signatures Associated with Severity of, Fatality of, and Recovery from COVID-19

**DOI:** 10.1128/spectrum.02194-22

**Published:** 2023-02-28

**Authors:** Luiz Gustavo Gardinassi, Carolina do Prado Servian, Gesiane da Silva Lima, Déborah Carolina Carvalho dos Anjos, Antonio Roberto Gomes Junior, Adriana Oliveira Guilarde, Moara Alves Santa Bárbara Borges, Gabriel Franco dos Santos, Brenda Grazielli Nogueira Moraes, João Marcos Maia Silva, Letícia Carrijo Masson, Flávia Pereira de Souza, Rodolfo Rodrigues da Silva, Giovanna Lopes de Araújo, Marcella Ferreira Rodrigues, Lidya Cardozo da Silva, Sueli Meira, Fabiola Souza Fiaccadori, Menira Souza, Pedro Roosevelt Torres Romão, Mônica Spadafora Ferreira, Verônica Coelho, Andréa Rodrigues Chaves, Rosineide Costa Simas, Boniek Gontijo Vaz, Simone Gonçalves Fonseca

**Affiliations:** a Departamento de Biociências e Tecnologia, Instituto de Patologia Tropical e Saúde Pública, Universidade Federal de Goiás, Goiânia, Goiás, Brazil; b Laboratório de Cromatografia e Espectrometria de Massas, Instituto de Química, Universidade Federal de Goiás, Goiânia, Goiás, Brazil; c Departamento de Medicina Tropical e Dermatologia, Instituto de Patologia Tropical e Saúde Pública, Universidade Federal de Goiás, Goiânia, Goiás, Brazil; d Hospital das Clínicas, Faculdade de Medicina, Universidade Federal de Goiás, Goiânia, Goiás, Brazil; e Laboratório Prof^a^ Margarida Dobler Komma, Instituto de Patologia Tropical e Saúde Pública, Universidade Federal de Goiás, Goiânia, Goiás, Brazil; f Laboratório de Imunologia Celular e Molecular, Programa de Pós-Graduação em Ciências da Saúde, Programa de Pós-Graduação em Ciências da Reabilitação, Universidade Federal de Ciências da Saúde de Porto Alegre, Porto Alegre, Rio Grande do Sul, Brazil; g Laboratório de Imunogenética, Instituto Butantan, São Paulo, São Paulo, Brazil; h Laboratório de Imunologia, Instituto do Coração, Faculdade de Medicina, Universidade de São Paulo, São Paulo, São Paulo, Brazil; i Laboratório de Histocompatibilidade e Imunidade Celular, Hospital das Clínicas HCFMUSP, Faculdade de Medicina, Universidade de São Paulo, São Paulo, São Paulo, Brazil; j Instituto de Investigação em Imunologia, Instituto Nacional de Ciências e Tecnologia, São Paulo, São Paulo, Brazil; University of Georgia

**Keywords:** COVID-19, data integration, inflammation, metabolomics

## Abstract

Severe manifestations of coronavirus disease 2019 (COVID-19) and mortality have been associated with physiological alterations that provide insights into the pathogenesis of the disease. Moreover, factors that drive recovery from COVID-19 can be explored to identify correlates of protection. The cellular metabolism represents a potential target to improve survival upon severe disease, but the associations between the metabolism and the inflammatory response during COVID-19 are not well defined. We analyzed blood laboratorial parameters, cytokines, and metabolomes of 150 individuals with mild to severe disease, of which 33 progressed to a fatal outcome. A subset of 20 individuals was followed up after hospital discharge and recovery from acute disease. We used hierarchical community networks to integrate metabolomics profiles with cytokines and markers of inflammation, coagulation, and tissue damage. Infection by severe acute respiratory syndrome coronavirus 2 (SARS-CoV-2) promotes significant alterations in the plasma metabolome, whose activity varies according to disease severity and correlates with oxygen saturation. Differential metabolism underlying death was marked by amino acids and related metabolites, such as glutamate, glutamyl-glutamate, and oxoproline, and lipids, including progesterone, phosphocholine, and lysophosphatidylcholines (lysoPCs). Individuals who recovered from severe disease displayed persistent alterations enriched for metabolism of purines and phosphatidylinositol phosphate and glycolysis. Recovery of mild disease was associated with vitamin E metabolism. Data integration shows that the metabolic response is a hub connecting other biological features during disease and recovery. Infection by SARS-CoV-2 induces concerted activity of metabolic and inflammatory responses that depend on disease severity and collectively predict clinical outcomes of COVID-19.

**IMPORTANCE** COVID-19 is characterized by diverse clinical outcomes that include asymptomatic to mild manifestations or severe disease and death. Infection by SARS-CoV-2 activates inflammatory and metabolic responses that drive protection or pathology. How inflammation and metabolism communicate during COVID-19 is not well defined. We used high-resolution mass spectrometry to investigate small biochemical compounds (<1,500 Da) in plasma of individuals with COVID-19 and controls. Age, sex, and comorbidities have a profound effect on the plasma metabolites of individuals with COVID-19, but we identified significant activity of pathways and metabolites related to amino acids, lipids, nucleotides, and vitamins determined by disease severity, survival outcome, and recovery. Furthermore, we identified metabolites associated with acute-phase proteins and coagulation factors, which collectively identify individuals with severe disease or individuals who died of severe COVID-19. Our study suggests that manipulating specific metabolic pathways can be explored to prevent hyperinflammation, organ dysfunction, and death.

## INTRODUCTION

The clinical spectrum of infection by severe acute respiratory syndrome coronavirus 2 (SARS-CoV-2) ranges from an asymptomatic state to various manifestations of coronavirus disease 2019 (COVID-19) and death. Despite the success of vaccination in preventing severe disease and mortality, a better understanding of the molecular mechanisms of COVID-19 pathogenesis is required to develop precise and accessible therapy. Factors associated with severe disease and death include age, sex, and comorbidities that seem to amplify viral replication and inflammatory responses (1, 2).

Inflammation is a critical component of severe COVID-19, and an immunological dysregulation is associated with unfavorable clinical outcomes (3). There is still limited knowledge about factors driving asymptomatic infection or mild disease. At the same time, post-COVID-19 survival and recovery have not been well characterized at the molecular level. Hematological and biochemical parameters as well as cytokines and chemokines, bioactive lipids, and metabolites are markedly modulated during SARS-CoV-2 infection and have been assessed for their diagnostic and prognostic potential (4–9). Specific cytokines, such as interleukin 6 (IL-6) and IL-1β, have been evaluated as therapeutic targets (10, 11). However, broad suppression of the inflammatory response via corticosteroid and JAK-STAT inhibitors has promoted better improvement than therapies targeting specific cytokine signaling (12, 13). Therefore, the concerted activity of multifactorial processes rather than signaling mediated by single candidate molecules could better explain the complexity of the pathophysiology of COVID-19 and its diverse clinical outcomes.

Cellular activation and function are tightly interwoven with metabolic adaptations, which support energetic demands, provide building blocks for catabolic or anabolic activity, and mediate cell signaling. Perturbations of homeostasis, such as infections and inflammation, can be characterized by metabolic fingerprints, which in turn provide information on molecular mechanisms of pathogenesis (14). High-resolution tandem mass spectrometry coupled with liquid chromatography (LC-MS/MS) is a powerful metabolomics platform to measure small molecules and lipids of <2,000 Da at large scales and has been helpful for dissecting metabolic profiles of blood from individuals with COVID-19 (15–17). However, metabolome-wide associations with inflammatory responses upon severe COVID-19, but especially death and recovery, are not well defined. Here, we performed an untargeted metabolomics study employing data-driven approaches to determine the metabolic profiles of individuals with COVID-19 and understand how they are associated with inflammation under diverse circumstances.

We found a significant influence of confounding factors, such as sex, age, and comorbidities, on the metabolomes of individuals with COVID-19. After controlling for these covariables, severity of disease still dictated the metabolic activity in the plasma, revealing metabolic pathways that also correlate with oxygen saturation. Modulation of glutamate, tryptophan, oxoproline, progesterone, lysophosphatidylcholines (lysoPCs) and related metabolites characterizes fatal disease, and patients who recover from acute COVID-19 display persistent metabolic alterations in the plasma. Importantly, metabolites are integrative hubs linking cytokines, inflammatory factors, and coagulation functions in multifactorial, multiscale networks that predict the severity of, fatality of, and recovery from COVID-19.

## RESULTS

### Demographic characteristics and inflammatory profiles of individuals with COVID-19.

We recruited 150 individuals who tested positive for SARS-CoV-2 by reverse transcription-quantitative PCR (RT-qPCR) or serology between June 2020 and February 2021. Twenty-seven individuals with negative RT-qPCR and serology for SARS-CoV-2 were enrolled as control donors. Individuals with COVID-19 were stratified into mild, moderate, and severe disease based on clinical parameters, including oxygen saturation and admission to an intensive care unit, in addition to those progressing to a fatal outcome (see Table S1 in the supplemental material). Longitudinal follow-up of a subset of individuals (*n* = 20) allowed paired sampling after recovery from COVID-19 (Table S2). The analysis of clinical profiles considering the individuals’ sex revealed a higher proportion of men with fatal COVID-19 (Table S1). The distribution of age was similar between controls and individuals with COVID-19, except for individuals with mild disease (Table S1). Demographic, clinical, and laboratorial data, including the description of comorbidities, symptoms, and treatments, are detailed in Tables S1 and S2.

Hematological characteristics and levels of biochemical laboratory parameters related to organ damage, acute-phase proteins, and coagulation functions were similar to those found in other cohorts (4, 18, 19). For example, individuals with fatal COVID-19 displayed lymphopenia, while individuals with mild disease and recovered individuals exhibited cell counts comparable to those of control donors (Fig. S1A). Neutrophil counts increased in individuals with moderate, severe, and fatal disease compared to control donors, with values in the group with fatal outcomes also differing from those in other groups (Fig. S1A). Levels of C-reactive protein (CRP), creatinine, and ferritin also increased in the plasma of individuals with fatal disease in comparison to almost all other groups (Fig. S1B). Relative abundance of most cytokines was elevated in the plasma of individuals with moderate, severe, and fatal COVID-19, with IL-6 reaching the highest levels among them (Fig. S1C). Compared to control donors, individuals who recovered from acute COVID-19 displayed small but significant alterations in the abundance of plasma cytokines (Fig. S1D).

### The metabolomes of individuals with COVID-19 are influenced by confounding factors.

**FIG 1:**
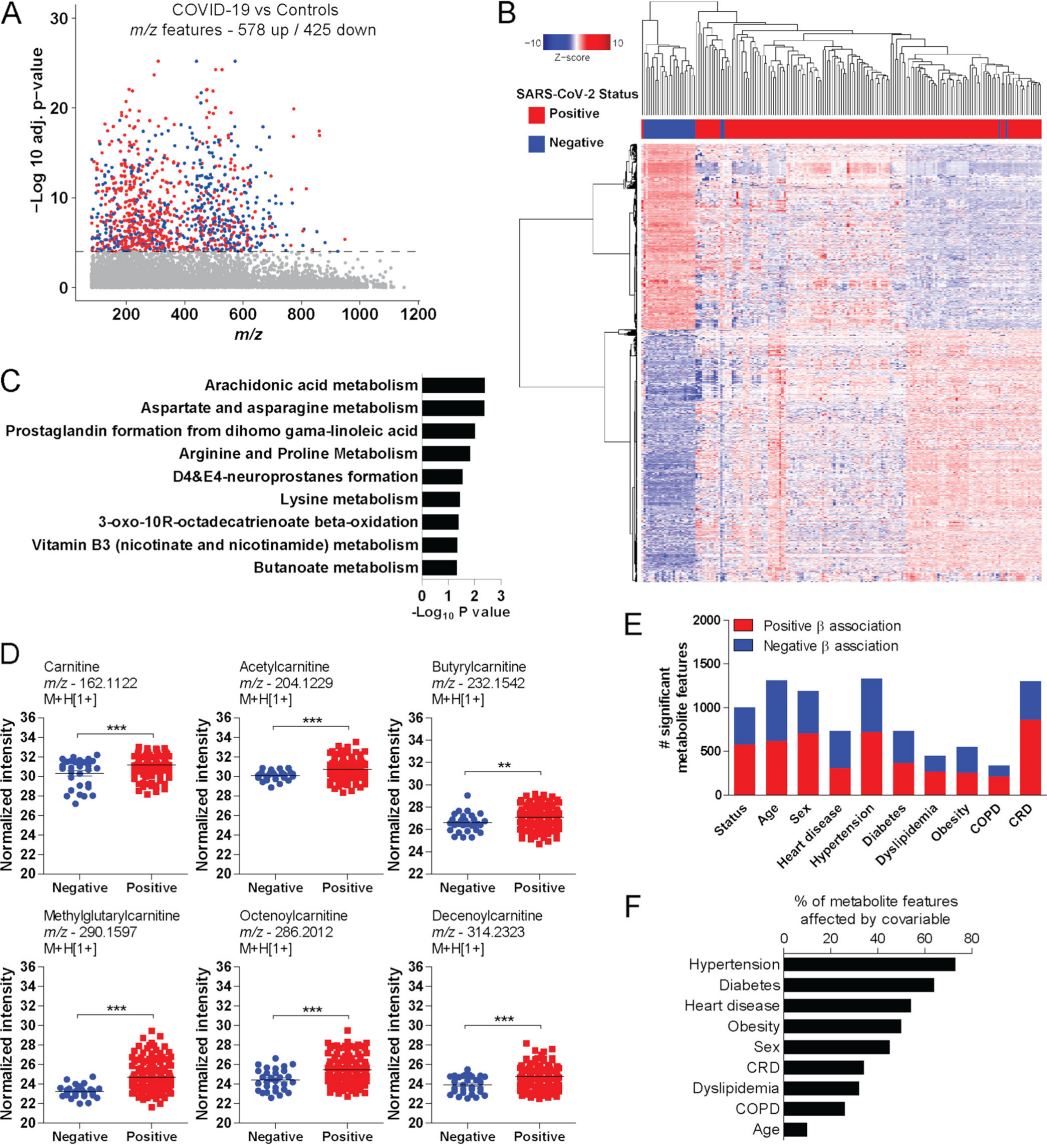
Plasma metabolomic signatures of SARS-CoV-2 infection. (A) Manhattan plot depicting significant metabolite features in plasma of individuals with COVID-19 compared to control donors. The dashed line indicates an FDR-adjusted *P* value of <0.0001. Upregulated features are in red and downregulated features in blue. (B) Two-way hierarchical clustering based on significant metabolite features. (C) Mummichog pathway analysis of significant features. (D) Differential abundance of carnitine and acylcarnitines according to infection status. (E) Number of significant metabolite features associated with SARS-CoV-2 infection, or age, sex, COPD, CRD, diabetes, dyslipidemia, heart disease, hypertension, and obesity. (F) Percentage of metabolite features whose coefficient of association with infection changes by more than 10% with the inclusion of the covariable in the logistic regression model. Tukey’s multiple-comparison test was used in additional statistics. **, *P* < 0.01; ***, *P* < 0.001.

To investigate the metabolic responses to SARS-CoV-2 infection, we performed an untargeted metabolomics analysis of plasma samples from individuals with COVID-19 and compared them to those from control donors. There were 578 up- and 425 downregulated metabolite features (false discovery rate [FDR] < 0.0001) in the plasma samples from individuals with COVID-19 (Fig. 1A). Hierarchical clustering based on significant features revealed two major clusters of individuals with positive or negative SARS-CoV-2 infection, except for a few outliers (Fig. 1B). Mummichog software analysis, which was designed to evaluated untargeted metabolomics data (20), predicted the activity of diverse metabolic pathways, including arachidonic acid metabolism and arginine and proline metabolism, among others that have been observed in independent cohorts of individuals with COVID-19 (Fig. 1C) (21). Among the significant metabolites, infection was associated with increased abundance of plasma carnitine, acetylcarnitine, butyrylcarnitine, methylglutarylcarnitine, octenoylcarnitine, and decenoylcarnitine (Fig. 1D).

The high heterogeneity of metabolomic signatures among individuals with COVID-19 suggests that intrinsic factors such as age or sex could affect levels of circulating metabolites and thus confound the results. To test whether these covariables could be confounding factors, we first performed logistic regressions to identify statistical associations between metabolite features and age, sex, heart disease, hypertension, diabetes, obesity, dyslipidemia, chronic renal disease (CRD), and chronic obstructive pulmonary disease (COPD) in our study cohort. All the tested variables were associated significantly (*P* < 0.05) with many metabolite features (Fig. 1E), while several metabolite features affected by SARS-CoV-2 infection were the same as those associated with covariables (Fig. S2). Because of the unbalanced number of individuals with comorbidities in our cohort (e.g., different sample sizes of individuals with diabetes and those with CRD), we used a different approach to understand the impact of covariables as confounding factors. We used logistic regression to calculate the metabolome-wide association with infection, comparing models that included the covariable of interest or not. For that, we calculated the difference in the measurement of association (regression coefficient) resulting from the two models and considered an effect if the difference was 10% or more for all metabolite features. We found that, to some extent, all the covariables influenced the plasma metabolome, where hypertension and diabetes were the major confounding variables (Fig. 1F). Therefore, the metabolomes of individuals with COVID-19 are influenced by factors beyond infection that can affect data interpretation.

### Differential metabolic activity according to COVID-19 severity.

**FIG 2:**
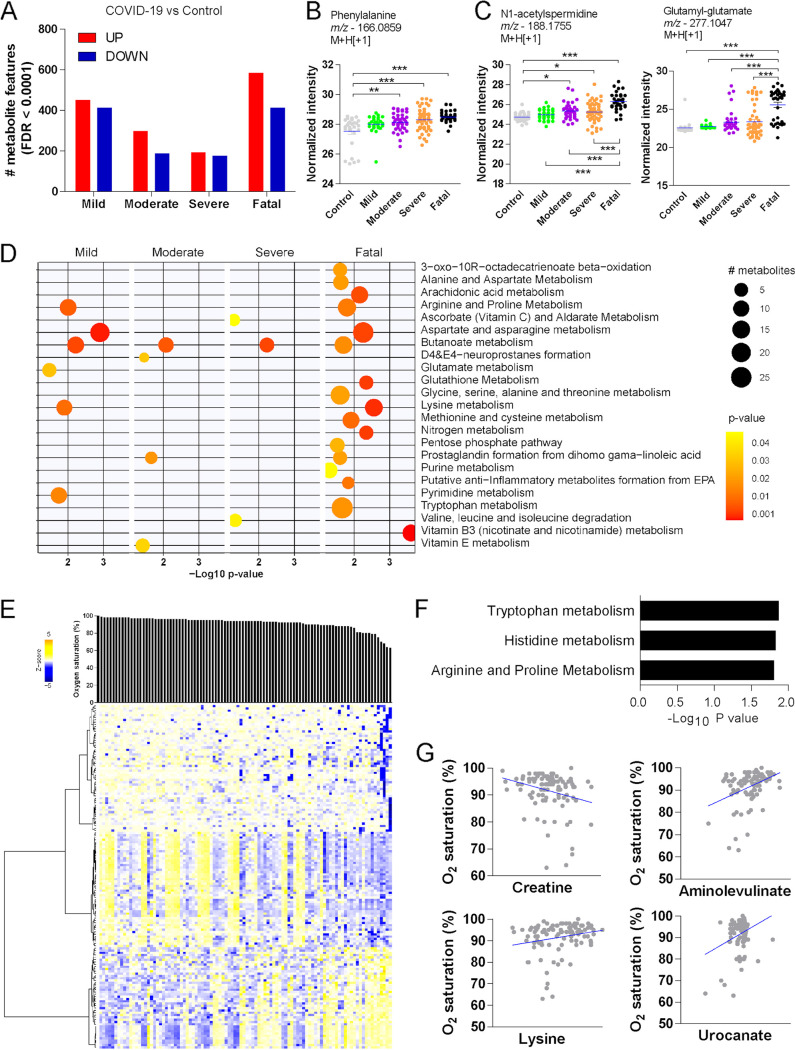
Metabolomic phenotypes according to severity of COVID-19. (A) Significant metabolite features between individuals with COVID-19 stratified by disease severity compared to control donors. (B) Differential abundance of phenylalanine. (C) Differential abundance of *N*1-acetylspermidine and glutamyl-glutamate according to disease severity. (D) Pathway enrichment analysis of significant metabolite features. (E) Hierarchical clustering based on significant metabolites associated with oxygen saturation (SpO_2_) in individuals with COVID-19. (F) Mummichog pathway analysis of metabolite features associated with SpO_2_. (G) Examples of metabolites associated with SpO_2_ (*P* < 0.05). Tukey’s multiple-comparison test was used in additional statistics. *, *P* < 0.05; **, *P* < 0. 01; ***, *P* < 0.001.

To identify metabolic correlates of disease severity, we compared individuals with mild, moderate, severe, or fatal COVID-19 to control donors, accounting for confounding factors in the linear regression models. The number of significant metabolite features (FDR < 0.0001) varied by severity classification, but individuals with fatal COVID-19 displayed the highest numbers of both up- and downregulated features (Fig. 2A). The abundance of carnitine (Fig. S3C) and phenylalanine (Fig. 2B) increased in the plasma of individuals with COVID-19 compared to controls but was similar among categories of severity. In agreement with previous findings (8), the abundance of *N*1-acetylspermidine increased with COVID-19, and it was even higher in the plasma of individuals with fatal outcomes (Fig. 2C). Fatal COVID-19 was also associated with increased abundance of glutamyl-glutamate in the plasma (Fig. 2C). Mummichog pathway analysis predicted the activity of several metabolic pathways according to each clinical stratification (Fig. 2D). As expected, fatal disease was associated with more pathways than the other categories of severity. Butanoate metabolism was significant for all groups of individuals with COVID-19 compared to control donors. Pathways such as arginine and proline metabolism, aspartate and asparagine metabolism, and lysine metabolism were enriched for mild and fatal disease, while prostaglandin formation from dihomo-γ-linoleic acid was enriched for moderated and fatal disease (Fig. 2D). Because of the significant effect of infection over the plasma metabolome, we then compared only the metabolomes of individuals with COVID-19. For that, we used a limma-moderated *F* test, which revealed 396 significant metabolite features (FDR < 0.0001) (Fig. S3A). Mummichog analysis predicted the activity of most of the same pathways when each group of individuals was compared to control donors (Fig. S3B).

Oxygen saturation (SpO_2_) is an important indicator of severity and has been used in clinical prediction scores (22, 23). We performed a metabolome-wide association analysis of SpO_2_ adjusted for confounding factors to identify metabolites predicting this clinical feature. We identified 149 significantly associated metabolite features (*P* < 0.001), of which 44 were negatively associated and 105 were positively associated with SpO_2_ in individuals with COVID-19 (Fig. 2E). Significant metabolites were predicted to be involved with tryptophan metabolism, histidine metabolism, and arginine and proline metabolism (Fig. 2F). At the metabolite level, an example of negative association includes creatine, while positive associations include aminolevulinate, lysine, and urocanate (Fig. 2G).

### Fatal COVID-19 is characterized by the modulation of lipids and amino acids.

**FIG 3:**
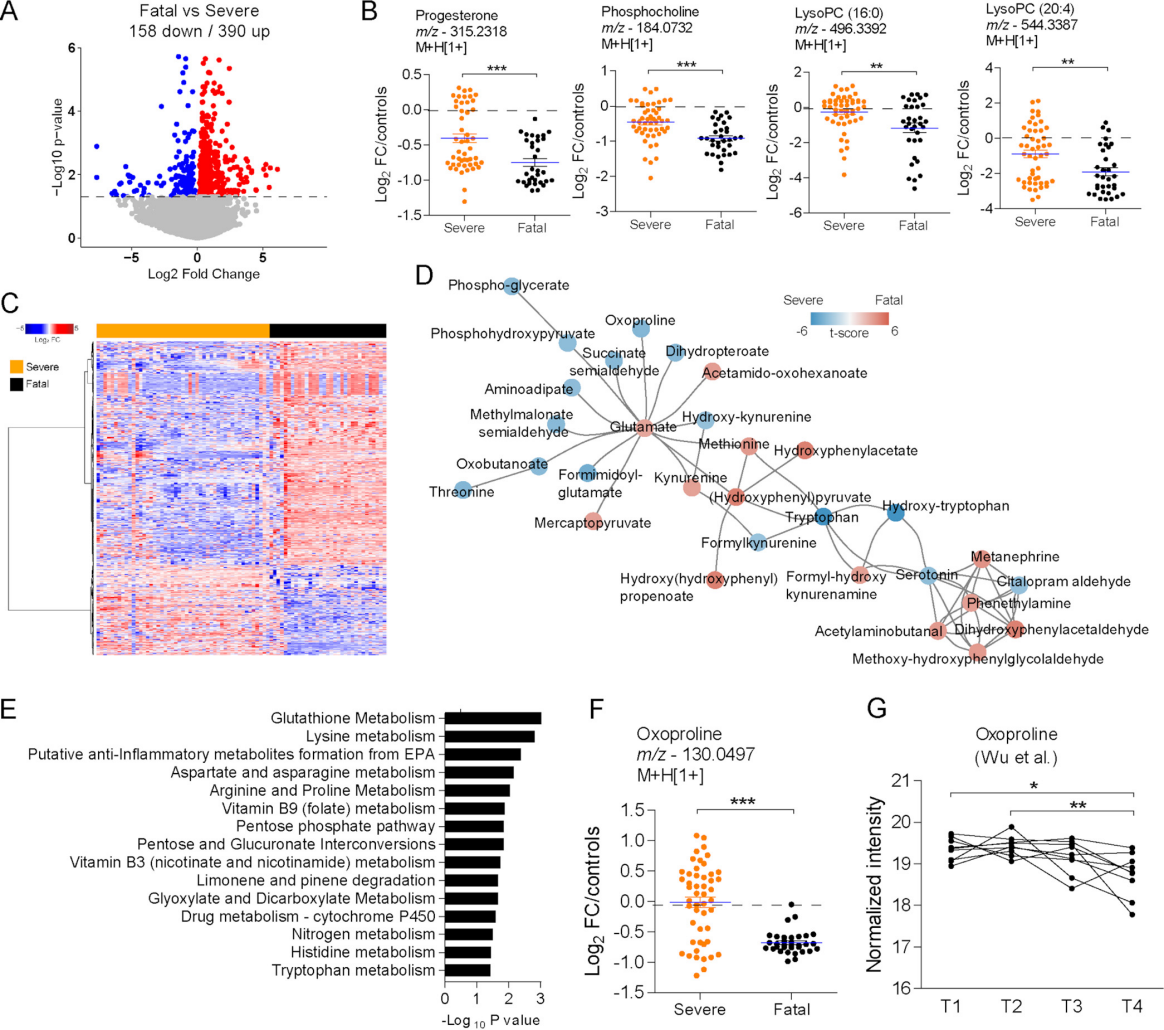
Metabolic correlates of fatal COVID-19. (A) Volcano plot demonstrating metabolite features differing in the plasma of individuals with severe or fatal COVID-19. The dashed line indicates an FDR of <0.05. (B) Differential abundance of progesterone, phosphocholine, lysoPC(16:0), and lysoPC(20:4) according to survival of severe disease or fatal outcome. (C) One-way hierarchical clustering based on significant metabolite features. (D) Mummichog modular analysis depicting a metabolic network of amino acids and derived metabolites. (E) Mummichog pathway analysis of significant metabolite features. (F) Differential abundance of oxoproline according to survival of severe disease or fatal outcome. (G) Reanalysis of the relative abundance of oxoproline along the course of 4 different time points before death due to COVID-19, retrieved from the study by Wu et al. (16). *, *P* < 0.05; **, *P* < 0.01; ***, *P* < 0.001.

To identify metabolites that are associated with fatal outcomes upon severe disease, we compared the metabolic activity between individuals with fatal disease and those that survived severe COVID-19. To account for the abundance from control donors before comparing the groups of COVID-19 patients, we subtracted the mean log_2_ intensity values of the control donors from those of COVID-19 samples. The resulting values reflect how much the abundance of metabolites from each individual in both groups changed relative to the mean abundance of control donors. The analysis revealed 158 down- and 393 upregulated metabolite features in the plasma of individuals with fatal disease (Fig. 3A). We detected reduced abundance of lipids such as progesterone, phosphocholine, lysoPC(16:0), and lysoPC(20:4) (Fig. 3B). Significant metabolite features show some heterogeneity in abundance among individuals who survived, which is less apparent in individuals with fatal outcome (Fig. 3C). Mummichog analysis predicted a significant metabolic network (*P* < 0.05) of amino acids and derived metabolites (Fig. 3D). Mummichog analysis also predicted activity of pathways such as glutathione metabolism, lysine metabolism, and arginine and proline metabolism (Fig. 3E). Levels of oxoproline (involved in glutathione metabolism) were reduced in the plasma of individuals with fatal COVID-19 (Fig. 3F). Reanalysis of longitudinal data from an independent cohort that progressed to a fatal outcome (16) demonstrates a gradual reduction in the abundance of plasma oxoproline with time (Fig. 3G).

### Integrated molecular networks associated with severity and fatality of COVID-19.

**FIG 4:**
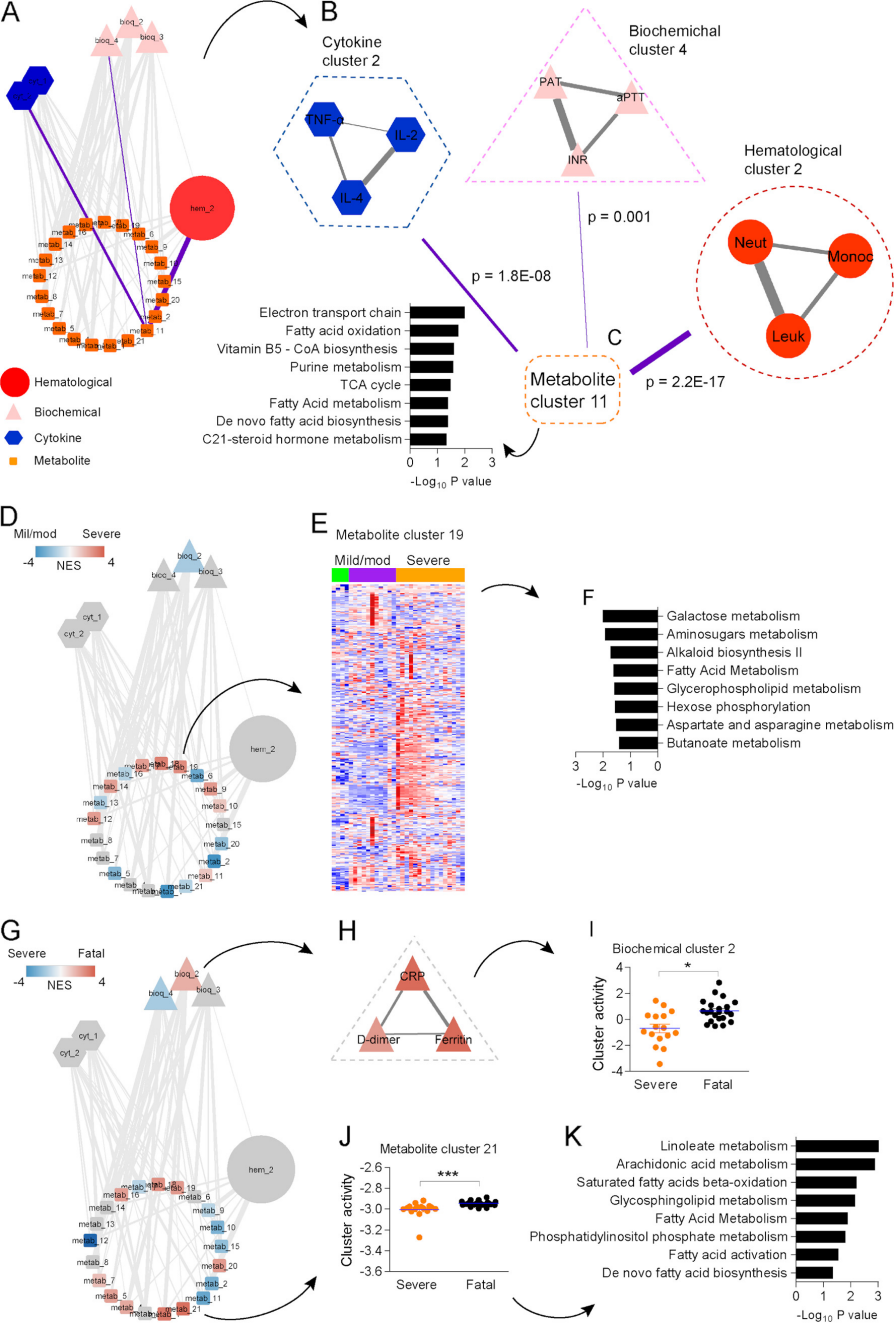
Integrative hierarchical community network underlying COVID-19. (A) The main network is composed of subnetworks (communities) of hematological data (hematological cluster), laboratory data (biochemical clusters), cytokine data (cytokine clusters) and metabolomics data (metabolite clusters), which are represented by nodes. Significant associations obtained via PLS regression and permutation test are represented by edges between nodes. (B) Amplified visualization of nodes linked by purple highlighted edges on the main network. (C) The most significant association in the network between metabolite cluster 11 and hematological cluster 2. (D) Communities related to the severity of COVID-19, colored by normalized enrichment score (NES) in a comparison between individuals with mild/moderate and severe COVID-19. Red indicates higher association with severe disease. (E) Heat map depicting the abundance of metabolite features composing metabolite cluster 19 in individuals with mild/moderate and severe disease. (F) Mummichog pathway analysis of metabolite cluster 19. (G) Communities related to fatality of COVID-19, colored by NES in a comparison between individuals with severe and fatal disease. Red indicates a higher association with death. (H) Amplified visualization of biochemical cluster 2. (I) Differential cluster activity of biochemical cluster 2 between individuals with severe and fatal disease. (J) Differential cluster activity of metabolite cluster 21 between individuals with severe disease and fatal disease. (K) Mummichog pathway analysis of metabolite cluster 21. *, *P* < 0.05; ***, *P* < 0.001.

Many studies demonstrate metabolites as key components of the host response to infection and vaccines (24–26), suggesting concerted activity of the metabolic and inflammatory responses during COVID-19. We used a hierarchical community network approach (24) to integrate hematological, acute-phase/coagulation, cytokine, and metabolomics data from 53 individuals with COVID-19 (Fig. S4). This method accounts for dimension reduction with meaningful grouping of features and different variance structures of distinct data types, because associations are tested via partial least-square (PLS) regression and significance is assessed by permutation, and therefore, only robust signals reach statistical significance (25). The leading network is composed of 27 nodes and 130 connections (edges at *P* < 0.05; 44 edges at *P* < 0.001) with 1 hematological cluster, 3 biochemical clusters, 2 cytokine clusters, and 21 metabolite clusters (Fig. 4A). Associations between a few nodes are highlighted in purple in Fig. 4A and enlarged in Fig. 4B, which shows that cytokine cluster 2 is composed of tumor necrosis factor alpha (TNF-α), IL-2 and IL-4; biochemical cluster 4 is composed of prothrombin activity time (PAT), activated partial thromboplastin time (aPTT), and international normalized ratio (INR); hematological cluster 2 is composed of neutrophils, monocytes, and total leukocytes; and metabolite cluster 11, which connects the different data types, is related to mitochondrial pathways, including electron transport chain, fatty acid oxidation, and tricarboxylic acid (TCA) cycle. The most significant association (*P* < 2.2E−17) was between hematological cluster 2 and metabolite cluster 11 (Fig. 4C), supporting our previous findings of increased mitochondrial activity in leukocytes of individuals with COVID-19 (27).

We queried the main network in search of subnetworks that could explain differences in disease severity and fatality of COVID-19. For that, we used the rank-based method GSEA (gene set enrichment analysis) using the network nodes as gene sets and permutation to test the significances. Comparison between individuals with mild or moderate and severe COVID-19 revealed biochemical cluster 2 and several metabolite clusters differentially associated with disease severity (Fig. 4D). Metabolite cluster 19 displayed many upregulated metabolites in plasma of individuals with severe disease (Fig. 4E), which are related to carbohydrate metabolism pathways such as galactose metabolism, amino sugar metabolism, and hexose phosphorylation (Fig. 4F). We also queried the leading network to identify clusters associated with fatal outcomes of COVID-19 (Fig. 4G). Biochemical cluster 2 (Fig. 4H) displayed increased activity in individuals with fatal disease (Fig. 4I). Furthermore, metabolite cluster 21 also exhibited increased activity in individuals with fatal COVID-19 (Fig. 4J) and is related to inflammatory lipid pathways, including linoleate metabolism and arachidonic acid metabolism, among others (Fig. 4K).

### COVID-19 induces persistent metabolic alterations in the blood.

To identify metabolic correlates of recovery from COVID-19, we followed up a subset of 20 individuals with varying clinical severity after recovery of acute disease. Compared to control donors, there were 493 significant metabolite features (FDR < 0.0001) in the plasma of recovered individuals (Fig. S5A). Predicted activity was related to pathways such as aspartate and asparagine metabolism, arginine and proline metabolism, and others (Fig. S5B). Of note, levels of adenine and guanine were higher in the plasma of recovered individuals, while the abundance of cytosine and spermidine decreased after recovery of acute COVID-19 (Fig. S5C).

**FIG 5:**
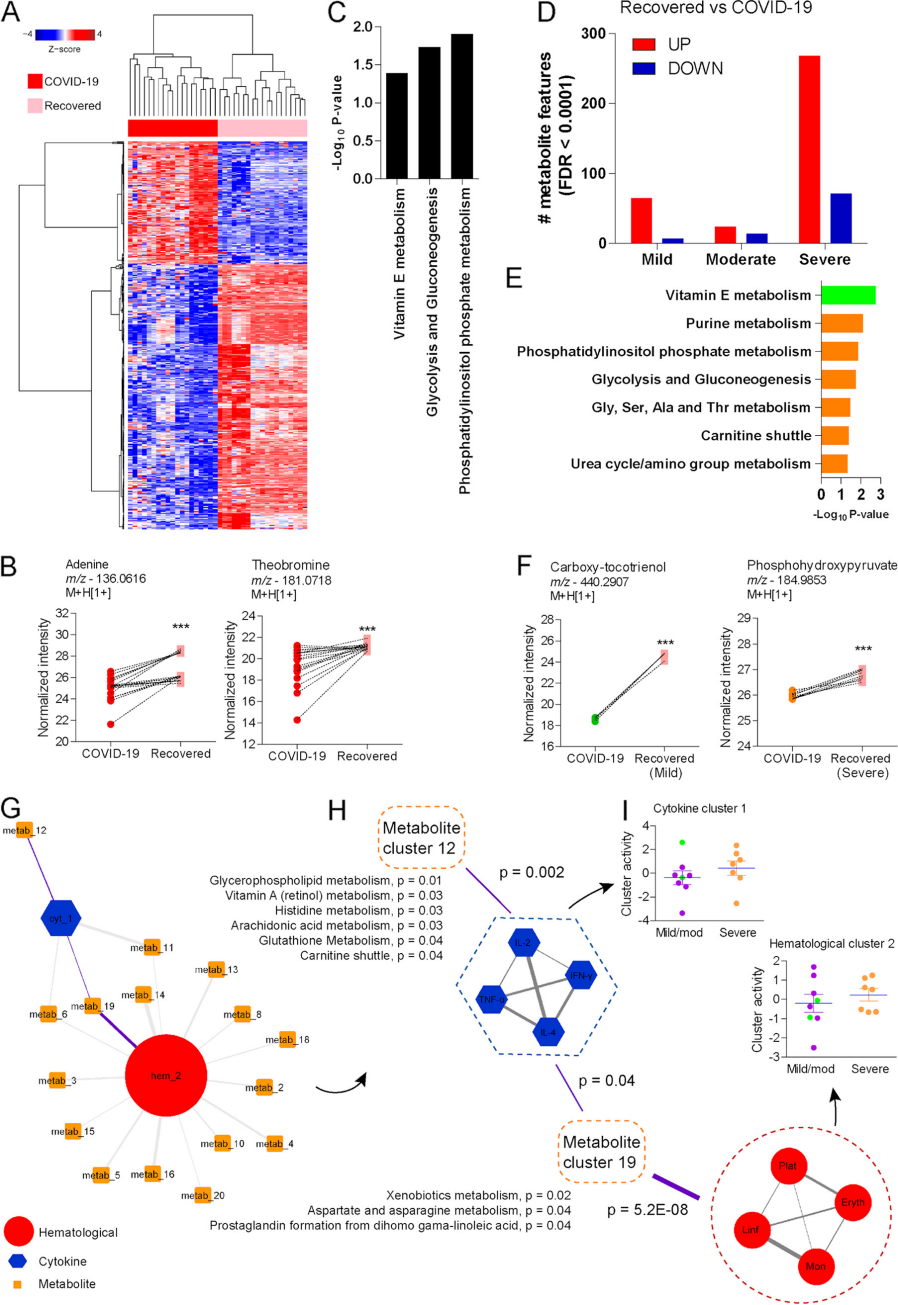
Metabolomic signatures of recovery from acute COVID-19. (A) Two-way hierarchical clustering of significant metabolite features on a longitudinal follow-up of selected individuals with COVID-19. (B) Differential abundance of adenine and theobromine before and after recovery from COVID-19. (C) Mummichog pathway analysis of significant metabolite features. (D) Significant metabolite features before and after recovery from acute COVID-19, stratified by disease severity. (E) Mummichog pathway analysis of significant metabolite features stratified by disease severity. (F) Differential abundance of metabolite features annotated as carboxy-tocotrienol and phosphohydroxypyruvate before and after recovery from acute COVID-19, stratified by disease severity. (G) Integrative hierarchical community network underlying recovery of acute COVID-19. (H) Amplified visualization of nodes linked by purple-highlighted edges on the main network. (I) Cluster activity of cytokine cluster 1 and hematological cluster 2 between patients with mild/moderate and severe disease who recovered from acute COVID-19. ***, *P* < 0.001.

Analysis of paired samples of the same group of individuals demonstrated 143 down- and 309 upregulated metabolite features (Fig. 5A), with significant increases in the abundance of metabolites such as adenine and theobromine after recovery from COVID-19 (Fig. 5B). Mummichog analysis predicted the activity of a few pathways, including phosphatidylinositol phosphate metabolism, glycolysis and gluconeogenesis, and vitamin E metabolism (Fig. 5C). Stratification by disease severity showed many significant features in the plasma of individuals who recovered from severe COVID-19 but not in those who recovered from mild or moderate disease (Fig. 5D). Pathway analysis predicted that activity of vitamin E metabolism is driven by recovery of mild disease (Fig. 5E, green bar), while other pathways such as purine metabolism, phosphatidylinositol phosphate metabolism, and glycolysis and gluconeogenesis are related to recovery of severe disease (Fig. 5E, orange bars). The abundance of the feature annotated as carboxy-tocotrienol increased in the plasma of individuals recovering from mild disease, while the feature annotated as phosphohydroxypyruvate increased in the plasma of individuals recovering from severe disease (Fig. 5F).

We used the hierarchical community network method to integrate hematological, cytokine, and metabolomics data from individuals who recovered from COVID-19. For this, we normalized data by subtracting measurements at time point 1 (disease) from those at time point 2 (recovery). Following, we applied hierarchical clustering followed by PLS regression to find associations, whose significance was tested on 1 million permutations. The network contains 18 nodes with 38 connections (at *P* < 0.05; 10 connections at *P* < 0.001) (Fig. 5G). Highlighted connections are displayed in Fig. 5H, showing that metabolite cluster 12 is enriched by glycerophospholipid metabolism or arachidonic acid metabolism and exhibits a single association with cytokine cluster 1. Cytokine and hematological clusters connect to metabolites, such as metabolite cluster 19, involved in xenobiotic metabolism or prostaglandin formation (Fig. 5H). Comparing the activities of cytokine and hematological clusters between individuals recovering from mild/moderate and severe disease revealed no differences (Fig. 5I). However, individuals recovering from severe COVID-19 exhibited reduced activity of metabolite cluster 1, which is related to steroid hormone biosynthesis, and metabolite cluster 4, which is related to phytanic acid oxidation (Fig. S6). In contrast, they displayed increased activity of metabolite cluster 7, which is related to drug metabolism, as well as metabolite cluster 13, which is associated with linoleate metabolism (Fig. S6).

## DISCUSSION

We employed an untargeted metabolomics approach to assess the molecular phenotypes in the plasma of individuals with mild to fatal COVID-19, and a subset that recovered from acute disease. Furthermore, we integrated biological factors that mediate and are affected by inflammation with the metabolic responses of individuals with COVID-19. This is important, because the cellular metabolism represents a potential target to modulate the inflammatory response, and therefore, there is great interest in discovering how metabolites impact COVID-19 (9, 15–17, 21, 28–33).

Our study demonstrates that infection by SARS-CoV-2 induces important adaptations in the metabolism that can be detected in the plasma. These alterations reflect the activity of cells in the upper and lower respiratory tracts but can also originate from other organs in response to inflammatory cues. Many of the detected metabolites and pathways are involved in immune and inflammatory responses. For instance, arachidonic acid metabolism and generation of eicosanoids are involved in diverse mechanisms of innate and adaptive immunity and have been explored during COVID-19 (34–36). In our study, arachidonic acid metabolism was mostly linked to death, suggesting eicosanoids as amplifiers of the inflammatory response. Furthermore, increased abundance of carnitine and acylcarnitines in plasma during COVID-19 suggests disruptions in mitochondrial activity and fatty acid oxidation and can be related to poorer clinical outcomes of overweight individuals with COVID-19 (37, 38). Other studies reported increased abundance of carnitine and/or acylcarnitines with infection by SARS-CoV-2, and many of them also associate this increase with disease severity (8, 39–42). In contrast, reduced levels of acylcarnitines were also implicated in COVID-19 severity (28, 43, 44). l-Carnitine may reduce the expression of receptors involved in SARS-CoV-2 entry into host cells and inhibit epithelial cell infection (45), and genetic traits inducing higher levels of l-carnitine are associated with reduced susceptibility and severity of COVID-19 (46). Although the regulation and function of carnitine and acylcarnitines during COVID-19 are still under debate, the current evidence points to crucial roles of these metabolites in infection and disease progression.

The infection by SARS-CoV-2 and severity of COVID-19 are associated with the activity of multiple metabolic pathways. However, fatal disease is associated with more perturbations in the plasma metabolome. Pathways such as lysine metabolism and tryptophan metabolism are also associated with levels of oxygen saturation, suggesting a role for amino acids and their derivatives in respiratory distress. In line with these findings, an independent metabolomics study reported altered amino acid catabolism in hypoxic individuals with COVID-19 (47). Hypoxic conditions, such as those observed in individuals with severe COVID-19, can impair mitochondrial function and cellular bioenergetics, causing energy deficits (48). As reported by independent studies, we observed increased abundance of phenylalanine with SARS-CoV-2 infection (32, 49, 50). Although differences between categories of severity did not reach statistical significance, phenylalanine has been implicated as a biomarker of COVID-19 severity (51–53).

The plasma metabolome of individuals with fatal COVID-19 was characterized by the differential activity of a metabolite network hinging on the amino acids glutamate and tryptophan. Many of the metabolites on the network have been previously associated with infection, sex disparities, and disease severity (7, 17, 28, 29, 54). In our study, fatal disease was associated with reduced abundance of oxoproline, which declined over time in an independent cohort of patients who also died due to COVID-19 (16). Oxoproline can be oxidized by oxoprolinase to glutamate, whose abundance was elevated in the plasma of individuals with fatal disease. In addition, levels of glutamyl-glutamate also increased in the plasma of patients who died. These metabolites are involved with glutathione metabolism, the most significant pathway differing between patients who survived or died after severe COVID-19. The balance between glutamate, oxoproline and glutamyl-amino acids can affect the synthesis of glutathione and tissue homeostasis, suggesting that failure in the generation and/or function of glutathione underlies fatal outcomes of COVID-19. Consistent with this hypothesis, SARS-CoV-2 infection impairs the metabolism of glutathione and its antioxidative function *in vitro* (55).

Of interest, our study suggests altered abundance of several lipids in the plasma of individuals with fatal disease, including progesterone, phosphocholine, and lysoPCs. Progesterone diffuses through cell membranes and binds to the progesterone receptor (PR) in the cytoplasm, where PR dimers form a complex that translocates to the nucleus, dampens inflammatory-gene transcription, and promotes expression of growth factors (56). Therefore, progesterone could regulate cellular responses of individuals with COVID-19 (57). At the same time, reproductive steroids may underlie sex disparities in clinical outcomes of COVID-19 (58). Importantly, a randomized, placebo-controlled trial demonstrated enhanced antibody production, reduced production of proinflammatory cytokines, and reduced severity in individuals with COVID-19 who were given estradiol and progesterone early in the course of the disease (59). LysoPCs upregulate adhesion molecules (60) and induce proinflammatory cytokines (61). Reduced levels of these lipids in fatal outcomes could reflect enhanced viral replication, as viruses alter the cell membrane composition during this process (62). In line with our findings, reduced levels of lysoPCs and other phospholipids predict the severity of COVID-19 (9).

Individuals with severe and fatal COVID-19 displayed neutrophilia and lymphopenia, as well as increased levels of CRP, ferritin, and cytokines such as IL-6, IL-10, gamma interferon (IFN-γ) and TNF-α, as described previously (4, 18, 19, 28). The integration of different orthogonal data in our study revealed that metabolite clusters associated with all other data types, but only one connection between hematological and a biochemical cluster reached statistical significance. These results suggest the metabolic response as an integrative hub for the communication between different physiological processes involved in the response to COVID-19. In association with many metabolite clusters, biochemical cluster 2, which includes CRP, D-dimer, and ferritin, predicted the fatality of individuals with COVID-19. Fatal outcome was also predicted by a cluster associated with inflammatory fatty acids (metabolite cluster 21), which emerged as critical molecules in COVID-19 pathogenesis (63, 64).

The understanding of metabolic profiles during convalescence and recovery from acute COVID-19 might reveal mechanisms of protection or even pathology associated with long COVID-19. There are several lines of evidence pointing for a return to baseline levels of metabolites affected by SARS-CoV-2 infection and disease severity, including acylcarnitines and lysoPCs (9, 41). However, metabolite abundance early (1 month) after recovery is still altered (65) and persists 2 to 3 months after nonsevere COVID-19 (41). Other studies evaluating individuals who recovered from more severe phenotypes also found persistent alterations in the plasma metabolome (17, 30, 32). Despite our small sample size, individuals who recovered from acute COVID-19 in our cohort also displayed persistent metabolic alterations. Paired samples from the same individuals revealed that patients with severe COVID-19 are the most affected. Example of altered metabolites include adenine, which inhibits the production of proinflammatory cytokines and bioactive lipids in cellular and animal models of inflammation (66, 67). The pathology of individuals recovering from severe COVID-19 may take longer to resolve, which in turn could stimulate prolonged production of anti-inflammatory metabolites. The metabolism of vitamin E was enriched in plasma of individuals who recovered from mild COVID-19. Vitamin E regulates the immune response and confers resistance to many viral infections (68). Furthermore, vitamin E and derived metabolites, such as tocotrienols, are antioxidant molecules that improve the response to oxidative stress (69) and also regulate inflammation (70). Of note, recovery of all individuals was coupled with higher levels of theobromine in the plasma. This metabolite is found in food and is endogenously metabolized from caffeine. Sickness behavior is characterized by reduced food ingestion, which affects host responses to infections (71), and theobromine levels might indicate improved nutritional status upon recovery or even contribute to the resolution of symptoms such as cough (72). Importantly, data integration revealed that metabolite clusters better explain differences in individuals who recovered from mild/moderate and severe disease.

Overall, the data indicate that metabolomic profiles of individuals with COVID-19 is influenced by a wide range of confounding factors, which were accounted for in our models. However, other factors were not controlled, including individual genetics, SARS-CoV-2 strain, diet, and drug therapy. We were unable to control for treatment, because individuals received different drugs via distinct regimens over the course of disease, including those with mild phenotypes, many of whom self-medicated. Our study is also limited to measurements of few cytokines and laboratorial and hematological parameters that do not reflect the entire complexity of the inflammatory response. Moreover, the small number of individuals followed up after hospital discharge may represent only individualized phenotypes. Importantly, the hypotheses raised by our study need to be validated in independent cohorts and mechanistic studies.

## MATERIALS AND METHODS

### Study population and sample processing.

Individuals with COVID-19 were admitted to the Hospital das Clínicas and Hospital das Clínicas de Campanha or recruited at the Laboratório Prof^a^ Margarida Dobler Komma at the Federal University of Goiás, Goiânia, Brazil between June 2020 and February 2021, before vaccination rollout. Blood samples were collected in EDTA tubes from 150 individuals who had SARS-CoV-2 infection confirmed by RT-qPCR test from nasopharyngeal swabs or by serological assays to detect specific IgM/IgG antibodies (Eco diagnostics) and from control donors (*n* = 27), who were negative for SARS-CoV-2 infection confirmed by RT-qPCR from nasopharyngeal swabs and serological IgM/IgG tests. Blood samples were collected an average of 13 days from symptom onset (Table S1). Paired blood samples were collected from a subset of 20 individuals with COVID-19 who were followed up after recovery on average of 172 days after symptom onset (Table S2). The criteria defined in the COVID-19 treatment guidelines (National Institutes of Health, USA) and the World Health Organization (73, 74) were used to stratify individuals with COVID-19 into mild disease (individuals presenting various signs and symptoms without shortness of breath, dyspnea, or abnormal chest imaging), moderate disease (individuals presenting radiologically confirmed pneumonitis, hospitalization, and oxygen therapy), severe disease (dyspnea, respiratory frequency of ≥30 breaths/min, oxygen saturation [SpO_2_] of ≤93%, and/or lung infiltrates of >50% within 24 to 48 h, including individuals who required monitoring and treatment in an intensive care unit and mechanical ventilation), or fatal COVID-19. Laboratory parameters of liver and kidney function, inflammatory markers and coagulation factors, red blood cells, hemoglobin, platelets, and total and differential leukocytes were determined using automated equipment and a hematology smear, respectively. Blood samples were centrifuged at 1,800 rpm for 10 min to obtain plasma, which was stored at −80°C. The research protocol was approved by Ethical Appreciation (CAAE: 30804220.2.0000.5078). All participants provided informed consent according to the regulations of the Human Ethical Committee at the Hospital das Clínicas, Faculdade de Medicina of Universidade Federal de Goiás (UFG-GO). For individuals in the intensive care unit and those who were unable to communicate, consent was obtained from a legally authorized representative (LAR).

### RNA extraction and detection of SARS-CoV-2.

Nasopharyngeal swab samples from individuals with COVID-19 or control donors were processed and analyzed by RT-qPCR. Briefly, RNA extraction was performed with the commercial Qiagen viral RNA minikit (Qiagen, Hilden, Germany), following the manufacturer’s instructions. The RNA extracts were subjected to RT-qPCR assay using the Promega Go-Taq Probe one-step RT-qPCR system, according to the manufacturer’s protocol. Primers, probe, and synthetic positive control (nCoVPC) were manufactured by IDT (Integrated DNA Technologies, Iowa, USA) and targeted two regions of the N gene (N1 and N2) and the endogenous control, human RNase P gene (RP).

### Cytokine measurements.

Plasma levels of the cytokines IL-2, IL-4, IL-6, IL-10, TNF, and IFN-γ were measured in samples using a BD cytometric bead array (CBA) human Th1/Th2 kit (BD Biosciences, San Jose, CA, USA), according to the manufacturer’s instructions. Briefly, after sample processing, the cytokine beads were counted using a flow cytometer (FACSCanto II; BD Biosciences, San Diego, CA, USA), and analyses were performed using FCAP Array (3.0) software (BD Biosciences, San Jose, CA, USA). Log_2_-transformed mean fluorescence intensities were used for statistical analyses and data integration.

### LC-MS/MS.

For metabolomics analyses, cold acetonitrile was added to plasma samples (2:1 [vol/vol]) and subjected to vortex mixing and centrifugation (10 min, 10,000 rpm at 4°C) for protein precipitation. The stable isotopes [^1^³C3]caffeine, [^15^N]tyrosine, and progesterone-d9 were used as internal standards, and samples were transferred to injecting vials for LC-MS/MS analysis, which was performed with a high-performance liquid chromatograph (HPLC-UV; 1220 Infinity; Agilent Technologies) coupled with a Q Exactive hybrid quadrupole-Orbitrap high-resolution mass spectrometer (Thermo Fisher). Reverse-phase C_18_ chromatography was performed with Zorbax Eclipse Plus C_18_ columns (4.6 by 150 mm; 3.5 μm; Agilent) and positive electrospray ionization. All samples were analyzed using a gradient elution program. The binary mobile phases were water–0.5% formic acid with 5 mM ammonium formate (A) and acetonitrile (B). Their gradient elution started with 20% B for 5 min, linearly increased to 100% B in 30 min, and was kept constant for 8 min at 100% B. The eluent was restored to the initial conditions in 4 min to re-equilibrate the column and held for the remaining 8 min. The flow rate was kept at 0.5 mL min^−1^. The injection volume for analysis was 3 μL, and the column temperature was set at 35°C. The electrospray ionization was operated with the following settings: spray voltage, 3.5 kV; capillary temperature, 269°C; S-lens RF level, 50 V; sheath gas flow rate, 53 L min^−1^; auxiliary gas flow rate, 14 L min^−1^; sweep gas flow rate, 3 L min^−1^. The high-resolution mass spectrometry was carried out in full MS/dd-MS2 mode. The mass range in the full MS scanning experiments was *m/z* 80 to 1,200. The max IT was set at 200 ms, and AGC target was set at 1 × 10^6^. For fragmentation acquisition, the top 5 (TopN, 5; loop count, 5) most abundant precursors were sequentially transferred into the C-Trap (AGC target 1 × 10^5^; max IT 50 ms) for collision. The collision energy for target analytes was 20, 30, and 35 eV. Resolving power was set at 140,000 and 70,000 for full MS and dd-MS2 acquisitions, respectively.

### Bioinformatics and statistical analyses.

Proteowizard software was used to convert .raw files into .mzXML format, and apLCMS R software (75) was used to perform peak deconvolution and detection, to filter noise, to align mass-to-charge ratio (*m/z*) and retention time, and to quantify metabolite features, which are defined by specific *m/z*, retention time, and intensity values for each sample. Pooled human plasma samples were used for quality control (QC) and included in every batch of samples. Replicate samples were summarized based on a Pearson correlation coefficient (*r*) of >0.7. Data were log_2_ transformed, and features were filtered out by 90% presence in all samples and a coefficient of variation of <0.2 based on QC samples, resulting in 9,893 metabolite features used in further analysis. We used the ComBat function of the sva R package to correct for batch and technical effects. The Mummichog software (version 2) was used to predict activity of metabolic pathways and networks (mass accuracy under 10 ppm) (20). The R package ggplot2 was used to generate volcano, bubble, and Manhattan plots. Heat maps were generated with gplots, and hierarchical clustering was performed with the amap package using Pearson correlation as the distance metric and ward linkage. MZmine v 2.5 software was used to process LC-MS/MS data, with noise set at 1E04 and 1E03 for the MS1 and MS2 levels, respectively. *m/z* tolerance was set at 0.02 *m/z* or 10 ppm. ADAP chromatogram builder parameters included the following: minimum group size in number of scans, 5; group intensity threshold, 5E04; and minimum highest intensity, 1E05. Deconvolution parameters included the following: minimum peak height, 1E05; peak duration, 0 to 3 min; and baseline level, 1E04. Isotopic grouping parameters included the following: RT tolerance, 0.25 min; maximum charge, 2; and representative isotope, most intense. Join aligner parameters included the following: weight for *m/z*, 75; RT tolerance, 0.25 min; and RT weight, 25. MS/MS patterns were exported, and fragment similarity searches were performed with METLIN Gen2 (https://metlincloud2.massconsortium.com/) (76) and MyCompoundID (http://www.mycompoundid.org/) (77) (Fig. S7).

Differential abundance was evaluated with moderated *t* test or moderated *F* test (analysis of variance [ANOVA]) using the limma R package, which enabled the design of models containing covariables to account for potential confounding factors, including age, sex, and comorbidities. Additional statistics included Tukeýs or Kruskal-Wallis multiple-comparison test. The influence of covariables including age, sex, and comorbidities over the metabolome was evaluated with logistic regression. Multivariable linear regression to identify association between metabolites and SpO_2_ included age, sex, and comorbidities as covariables. Regressions were performed with glm function of the epicalc R package. FDR was calculated with the Benjamini-Hochberg method.

Data integration was performed using the hierarchical community network approach using python, as described previously (25, 26). Unsupervised hierarchical clustering based on correlation metrics was used to collapse hematological, laboratory, and cytokine data into clusters. For metabolite features, the same hierarchical clustering was applied, but close retention time was enforced within clusters to group different ions reflecting the same metabolite (25). PLS regression was used to calculate associations between clusters, while the significance (*P* value) of such associations was computed on over 1 million permutations for each pair of data, resampling both samples and features. Each node is a subnetwork, which enables an overview of the leading network and subnetworks for more details at multiple zoom levels. To query the main network, we used t-scores as a primary score in GSEA software. t-scores were obtained from limma output comparing individuals with mild/moderate and severe disease or comparing individuals with severe and fatal COVID-19. To integrate data from recovered individuals, we normalized the data by subtracting values for time point 1 (disease) from those for time point 2 (recovery), whereas biochemical clusters were not included, because many laboratory parameters were unavailable upon recovery. The networks of significantly associated clusters were visualized with Cytoscape v. 3.8.2. Activity of each cluster on a per-sample basis was calculated as follows: sum(*z* score)/square root(number of metabolite features).

### Data availability.

Metabolomics data have been deposited in the Metabolomics Workbench database with the identifier ST002291. The processed feature table used in the analyses is available at Figshare (https://doi.org/10.6084/m9.figshare.22047761.v1). Phenotypic data and annotations are provided in Data Set S1. Code used in the statistical analyses of metabolomics data is provided in Data Set S2.

## Supplementary Material

Reviewer comments

## References

[1] Santos E Silva JC, Vasconcelos AP, Noma IHY, Noronha NY, Aquino R, Giddaluru J, Durão L, Costa-Martins AG, Schuch V, Moraes-Vieira PM, Nakaya HI. 2021. Gene signatures of autopsy lungs from obese patients with COVID-19. Clin Nutr ESPEN 44:475–478. doi:10.1016/j.clnesp.2021.05.004. 34330510 PMC8149170

[2] Freire PP, Marques AH, Baiocchi GC, Schimke LF, Fonseca DL, Salgado RC, Filgueiras IS, Napoleao SM, Plaça DR, Akashi KT, Hirata TDC, El Khawanky N, Giil LM, Cabral-Miranda G, Carvalho RF, Ferreira LCS, Condino-Neto A, Nakaya HI, Jurisica I, Ochs HD, Camara NOS, Calich VLG, Cabral-Marques O. 2021. The relationship between cytokine and neutrophil gene network distinguishes SARS-CoV-2-infected patients by sex and age. JCI Insight 6:147535. doi:10.1172/jci.insight.147535. 34027897 PMC8262322

[3] Lucas C, Wong P, Klein J, Castro TBR, Silva J, Sundaram M, Ellingson MK, Mao T, Oh JE, Israelow B, Takahashi T, Tokuyama M, Lu P, Venkataraman A, Park A, Mohanty S, Wang H, Wyllie AL, Vogels CBF, Earnest R, Lapidus S, Ott IM, Moore AJ, Muenker MC, Fournier JB, Campbell M, Odio CD, Casanovas-Massana A, Herbst R, Shaw AC, Medzhitov R, Schulz WL, Grubaugh ND, Dela Cruz C, Farhadian S, Ko AI, Omer SB, Iwasaki A. Yale IMPACT Team. 2020. Longitudinal analyses reveal immunological misfiring in severe COVID-19. Nature 584:463–469. doi:10.1038/s41586-020-2588-y. 32717743 PMC7477538

[4] ten-Caten F, Gonzalez-Dias P, Castro Í, Ogava RLT, Giddaluru J, Silva JCS, Martins F, Gonçalves ANA, Costa-Martins AG, Araujo JD, Viegas AC, Cunha FQ, Farsky S, Bozza FA, Levin AS, Pannaraj PS, de Silva TI, Minoprio P, da Silva FP, Andrade BB, Nakaya HI. 2021. In-depth analysis of laboratory parameters reveals the interplay between sex, age, and systemic inflammation in individuals with COVID-19. Int J Infect Dis 105:579–587. doi:10.1016/j.ijid.2021.03.016. 33713813 PMC7944763

[5] Del Valle DM, Kim-Schulze S, Huang H-H, Beckmann ND, Nirenberg S, Wang B, Lavin Y, Swartz TH, Madduri D, Stock A, Marron TU, Xie H, Patel M, Tuballes K, Van Oekelen O, Rahman A, Kovatch P, Aberg JA, Schadt E, Jagannath S, Mazumdar M, Charney AW, Firpo-Betancourt A, Mendu DR, Jhang J, Reich D, Sigel K, Cordon-Cardo C, Feldmann M, Parekh S, Merad M, Gnjatic S. 2020. An inflammatory cytokine signature predicts COVID-19 severity and survival. Nat Med 26:1636–1643. doi:10.1038/s41591-020-1051-9. 32839624 PMC7869028

[6] Hao Y, Zhang Z, Feng G, Chen M, Wan Q, Lin J, Wu L, Nie W, Chen S. 2021. Distinct lipid metabolic dysregulation in asymptomatic COVID-19. iScience 24:102974. doi:10.1016/j.isci.2021.102974. 34396083 PMC8356725

[7] Danlos F-X, Grajeda-Iglesias C, Durand S, Sauvat A, Roumier M, Cantin D, Colomba E, Rohmer J, Pommeret F, Baciarello G, Willekens C, Vasse M, Griscelli F, Fahrner J-E, Goubet A-G, Dubuisson A, Derosa L, Nirmalathasan N, Bredel D, Mouraud S, Pradon C, Stoclin A, Rozenberg F, Duchemin J, Jourdi G, Ellouze S, Levavasseur F, Albigès L, Soria J-C, Barlesi F, Solary E, André F, Pène F, Ackerman F, Mouthon L, Zitvogel L, Marabelle A, Michot J-M, Fontenay M, Kroemer G. 2021. Metabolomic analyses of COVID-19 patients unravel stage-dependent and prognostic biomarkers. Cell Death Dis 12:1–11. doi:10.1038/s41419-021-03540-y. 33707411 PMC7948172

[8] Roberts I, Wright Muelas M, Taylor JM, Davison AS, Xu Y, Grixti JM, Gotts N, Sorokin A, Goodacre R, Kell DB. 2021. Untargeted metabolomics of COVID-19 patient serum reveals potential prognostic markers of both severity and outcome. Metabolomics 18:6. doi:10.1007/s11306-021-01859-3. 34928464 PMC8686810

[9] Sindelar M, Stancliffe E, Schwaiger-Haber M, Anbukumar DS, Adkins-Travis K, Goss CW, O'Halloran JA, Mudd PA, Liu W-C, Albrecht RA, García-Sastre A, Shriver LP, Patti GJ. 2021. Longitudinal metabolomics of human plasma reveals prognostic markers of COVID-19 disease severity. Cell Rep Med 2:100369. doi:10.1016/j.xcrm.2021.100369. 34308390 PMC8292035

[10] Tharaux P-L, Pialoux G, Pavot A, Mariette X, Hermine O, Resche-Rigon M, Porcher R, Ravaud P, Bureau S, Dougados M, Tibi A, Azoulay E, Cadranel J, Emmerich J, Fartoukh M, Guidet B, Humbert M, Lacombe K, Mahevas M, Pene F, Pourchet-Martinez V, Schlemmer F, Yazdanpanah Y, Baron G, Perrodeau E, Vanhoye D, Kedzia C, Demerville L, Gysembergh-Houal A, Bourgoin A, Dalibey S, Raked N, Mameri L, Alary S, Hamiria S, Bariz T, Semri H, Hai DM, Benafla M, Belloul M, Vauboin P, Flamand S, Pacheco C, Walter-Petrich A, Stan E, Benarab S, Nyanou C, Montlahuc C, Biard L, Charreteur R, et al. 2021. Effect of anakinra versus usual care in adults in hospital with COVID-19 and mild-to-moderate pneumonia (CORIMUNO-ANA-1): a randomised controlled trial. Lancet Respir Med 9:295–304. doi:10.1016/S2213-2600(20)30556-7. 33493450 PMC7825875

[11] Gordon AC, Mouncey PR, Al-Beidh F, Rowan KM, Nichol AD, Arabi YM, Annane D, Beane A, van Bentum-Puijk W, Berry LR, Bhimani Z, Bonten MJM, Bradbury CA, Brunkhorst FM, Buzgau A, Cheng AC, Detry MA, Duffy EJ, Estcourt LJ, Fitzgerald M, Goossens H, Haniffa R, Higgins AM, Hills TE, Horvat CM, Lamontagne F, Lawler PR, Leavis HL, Linstrum KM, Litton E, Lorenzi E, Marshall JC, Mayr FB, McAuley DF, McGlothlin A, McGuinness SP, McVerry BJ, Montgomery SK, Morpeth SC, Murthy S, Orr K, Parke RL, Parker JC, Patanwala AE, Pettilä V, Rademaker E, Santos MS, Saunders CT, Seymour CW, Shankar-Hari M. REMAP-CAP Investigators, et al. 2021. Interleukin-6 receptor antagonists in critically ill patients with Covid-19. N Engl J Med 384:1491–1502. doi:10.1056/NEJMoa2100433. 33631065 PMC7953461

[12] Horby P, Lim WS, Emberson JR, Mafham M, Bell JL, Linsell L, Staplin N, Brightling C, Ustianowski A, Elmahi E, Prudon B, Green C, Felton T, Chadwick D, Rege K, Fegan C, Chappell LC, Faust SN, Jaki T, Jeffery K, Montgomery A, Rowan K, Juszczak E, Baillie JK, Haynes R, Landray MJ. RECOVERY Collaborative Group. 2021. Dexamethasone in hospitalized patients with Covid-19. N Engl J Med 384:693–704. doi:10.1056/NEJMoa2021436. 32678530 PMC7383595

[13] Guimarães PO, Quirk D, Furtado RH, Maia LN, Saraiva JF, Antunes MO, Kalil Filho R, Junior VM, Soeiro AM, Tognon AP, Veiga VC, Martins PA, Moia DDF, Sampaio BS, Assis SRL, Soares RVP, Piano LPA, Castilho K, Momesso R, Monfardini F, Guimarães HP, Ponce de Leon D, Dulcine M, Pinheiro MRT, Gunay LM, Deuring JJ, Rizzo LV, Koncz T, Berwanger O. STOP-COVID Trial Investigators. 2021. Tofacitinib in patients hospitalized with Covid-19 pneumonia. N Engl J Med 385:406–415. doi:10.1056/NEJMoa2101643. 34133856 PMC8220898

[14] Sinclair C, Bommakanti G, Gardinassi L, Loebbermann J, Johnson MJ, Hakimpour P, Hagan T, Benitez L, Todor A, Machiah D, Oriss T, Ray A, Bosinger S, Ravindran R, Li S, Pulendran B. 2017. mTOR regulates metabolic adaptation of APCs in the lung and controls the outcome of allergic inflammation. Science 357:1014–1021. doi:10.1126/science.aaj2155. 28798047 PMC5746055

[15] Shen B, Yi X, Sun Y, Bi X, Du J, Zhang C, Quan S, Zhang F, Sun R, Qian L, Ge W, Liu W, Liang S, Chen H, Zhang Y, Li J, Xu J, He Z, Chen B, Wang J, Yan H, Zheng Y, Wang D, Zhu J, Kong Z, Kang Z, Liang X, Ding X, Ruan G, Xiang N, Cai X, Gao H, Li L, Li S, Xiao Q, Lu T, Zhu Y, Liu H, Chen H, Guo T. 2020. Proteomic and metabolomic characterization of COVID-19 patient sera. Cell 182:59–72.E15. doi:10.1016/j.cell.2020.05.032. 32492406 PMC7254001

[16] Wu D, Shu T, Yang X, Song J-X, Zhang M, Yao C, Liu W, Huang M, Yu Y, Yang Q, Zhu T, Xu J, Mu J, Wang Y, Wang H, Tang T, Ren Y, Wu Y, Lin S-H, Qiu Y, Zhang D-Y, Shang Y, Zhou X. 2020. Plasma metabolomic and lipidomic alterations associated with COVID-19. Natl Sci Rev 7:1157–1168. doi:10.1093/nsr/nwaa086. 34676128 PMC7197563

[17] Jia H, Liu C, Li D, Huang Q, Liu D, Zhang Y, Ye C, Zhou D, Wang Y, Tan Y, Li K, Lin F, Zhang H, Lin J, Xu Y, Liu J, Zeng Q, Hong J, Chen G, Zhang H, Zheng L, Deng X, Ke C, Gao Y, Fan J, Di B, Liang H. 2022. Metabolomic analyses reveal new stage-specific features of COVID-19. Eur Respir J 59:2100284. doi:10.1183/13993003.00284-2021. 34289974 PMC8311281

[18] Du Y, Tu L, Zhu P, Mu M, Wang R, Yang P, Wang X, Hu C, Ping R, Hu P, Li T, Cao F, Chang C, Hu Q, Jin Y, Xu G. 2020. Clinical features of 85 fatal cases of COVID-19 from Wuhan. a retrospective observational study. Am J Respir Crit Care Med 201:1372–1379. doi:10.1164/rccm.202003-0543OC. 32242738 PMC7258652

[19] Guan W-J, Ni Z-Y, Hu Y, Liang W-H, Ou C-Q, He J-X, Liu L, Shan H, Lei C-L, Hui DSC, Du B, Li L-J, Zeng G, Yuen K-Y, Chen R-C, Tang C-L, Wang T, Chen P-Y, Xiang J, Li S-Y, Wang J-L, Liang Z-J, Peng Y-X, Wei L, Liu Y, Hu Y-H, Peng P, Wang J-M, Liu J-Y, Chen Z, Li G, Zheng Z-J, Qiu S-Q, Luo J, Ye C-J, Zhu S-Y, Zhong N-S. China Medical Treatment Expert Group for Covid-19. 2020. Clinical characteristics of coronavirus disease 2019 in China. N Engl J Med 382:1708–1720. doi:10.1056/NEJMoa2002032. 32109013 PMC7092819

[20] Li S, Park Y, Duraisingham S, Strobel FH, Khan N, Soltow QA, Jones DP, Pulendran B. 2013. Predicting network activity from high throughput metabolomics. PLoS Comput Biol 9:e1003123. doi:10.1371/journal.pcbi.1003123. 23861661 PMC3701697

[21] Pang Z, Zhou G, Chong J, Xia J. 2021. Comprehensive meta-analysis of COVID-19 global metabolomics datasets. Metabolites 11:44. doi:10.3390/metabo11010044. 33435351 PMC7827862

[22] Alkaabi S, Alnuaimi A, Alharbi M, Amari MA, Ganapathy R, Iqbal I, Nauman J, Oulhaj A. 2021. A clinical risk score to predict in-hospital mortality in critically ill patients with COVID-19: a retrospective cohort study. BMJ Open 11:e048770. doi:10.1136/bmjopen-2021-048770. PMC8392735 34446489

[23] Xu J, Wang W, Ye H, Pang W, Pang P, Tang M, Xie F, Li Z, Li B, Liang A, Zhuang J, Yang J, Zhang C, Ren J, Tian L, Li Z, Xia J, Gale RP, Shan H, Liang Y. 2021. A predictive score for progression of COVID-19 in hospitalized persons: a cohort study. NPJ Prim Care Respir Med 31:33. doi:10.1038/s41533-021-00244-w. 34083541 PMC8175565

[24] Li S, Sullivan NL, Rouphael N, Yu T, Banton S, Maddur MS, McCausland M, Chiu C, Canniff J, Dubey S, Liu K, Tran V, Hagan T, Duraisingham S, Wieland A, Mehta AK, Whitaker JA, Subramaniam S, Jones DP, Sette A, Vora K, Weinberg A, Mulligan MJ, Nakaya HI, Levin M, Ahmed R, Pulendran B. 2017. Metabolic phenotypes of response to vaccination in humans. Cell 169:862–877.E17. doi:10.1016/j.cell.2017.04.026. 28502771 PMC5711477

[25] Gardinassi LG, Arévalo-Herrera M, Herrera S, Cordy RJ, Tran V, Smith MR, Johnson MS, Chacko B, Liu KH, Darley-Usmar VM, Go Y-M, Jones DP, Galinski MR, Li S. MaHPIC Consortium. 2018. Integrative metabolomics and transcriptomics signatures of clinical tolerance to Plasmodium vivax reveal activation of innate cell immunity and T cell signaling. Redox Biol 17:158–170. doi:10.1016/j.redox.2018.04.011. 29698924 PMC6007173

[26] Hagan T, Cortese M, Rouphael N, Boudreau C, Linde C, Maddur MS, Das J, Wang H, Guthmiller J, Zheng N-Y, Huang M, Uphadhyay AA, Gardinassi L, Petitdemange C, McCullough MP, Johnson SJ, Gill K, Cervasi B, Zou J, Bretin A, Hahn M, Gewirtz AT, Bosinger SE, Wilson PC, Li S, Alter G, Khurana S, Golding H, Pulendran B. 2019. Antibiotics-driven gut microbiome perturbation alters immunity to vaccines in humans. Cell 178:1313–1328.E13. doi:10.1016/j.cell.2019.08.010. 31491384 PMC6750738

[27] Gardinassi LG, Souza COS, Sales-Campos H, Fonseca SG. 2020. Immune and metabolic signatures of COVID-19 revealed by transcriptomics data reuse. Front Immunol 11:1636. doi:10.3389/fimmu.2020.01636. 32670298 PMC7332781

[28] Thomas T, Stefanoni D, Reisz JA, Nemkov T, Bertolone L, Francis RO, Hudson KE, Zimring JC, Hansen KC, Hod EA, Spitalnik SL, D’Alessandro A. 2020. COVID-19 infection alters kynurenine and fatty acid metabolism, correlating with IL-6 levels and renal status. JCI Insight 5:140327. doi:10.1172/jci.insight.140327. 32559180 PMC7453907

[29] Xiao N, Nie M, Pang H, Wang B, Hu J, Meng X, Li K, Ran X, Long Q, Deng H, Chen N, Li S, Tang N, Huang A, Hu Z. 2021. Integrated cytokine and metabolite analysis reveals immunometabolic reprogramming in COVID-19 patients with therapeutic implications. Nat Commun 12:1618. doi:10.1038/s41467-021-21907-9. 33712622 PMC7955129

[30] Xu J, Zhou M, Luo P, Yin Z, Wang S, Liao T, Yang F, Wang Z, Yang D, Peng Y, Geng W, Li Y, Zhang H, Jin Y. 2021. Plasma metabolomic profiling of patients recovered from coronavirus disease 2019 (COVID-19) with pulmonary sequelae 3 months after discharge. Clin Infect Dis 73:2228–2239. doi:10.1093/cid/ciab147. 33596592 PMC7929060

[31] Lorente JA, Nin N, Villa P, Vasco D, Miguel-Coello AB, Rodriguez I, Herrero R, Peñuelas O, Ruiz-Cabello J, Izquierdo-Garcia JL. 2021. Metabolomic diferences between COVID-19 and H1N1 influenza induced ARDS. Crit Care 25:390. doi:10.1186/s13054-021-03810-3. 34781986 PMC8591432

[32] Ansone L, Briviba M, Silamikelis I, Terentjeva A, Perkons I, Birzniece L, Rovite V, Rozentale B, Viksna L, Kolesova O, Klavins K, Klovins J. 2021. Amino acid metabolism is significantly altered at the time of admission in hospital for severe COVID-19 patients: findings from longitudinal targeted metabolomics analysis. Microbiol Spectr 9:e00338-21. doi:10.1128/spectrum.00338-21. 34878333 PMC8653833

[33] Oliveira LB, Mwangi VI, Sartim MA, Delafiori J, Sales GM, de Oliveira AN, Busanello ENB, de Val FFAE, Xavier MS, Costa FT, Baía-da-Silva DC, de Sampaio VS, de Lacerda MVG, Monteiro WM, Catharino RR, de Melo GC. 2022. Metabolomic profiling of plasma reveals differential disease severity markers in COVID-19 patients. Front Microbiol 13:844283. doi:10.3389/fmicb.2022.844283. 35572676 PMC9094083

[34] Zaid Y, Doré É, Dubuc I, Archambault A-S, Flamand O, Laviolette M, Flamand N, Boilard É, Flamand L. 2021. Chemokines and eicosanoids fuel the hyperinflammation within the lungs of patients with severe COVID-19. J Allergy Clin Immunol 148:368–380.E3. doi:10.1016/j.jaci.2021.05.032. 34111453 PMC8180473

[35] Schwarz B, Sharma L, Roberts L, Peng X, Bermejo S, Leighton I, Casanovas-Massana A, Minasyan M, Farhadian S, Ko AI, Dela Cruz CS, Bosio CM. Yale IMPACT Team. 2021. Cutting edge: severe SARS-CoV-2 infection in humans is defined by a shift in the serum lipidome, resulting in dysregulation of eicosanoid immune mediators. J Immunol 206:329–334. doi:10.4049/jimmunol.2001025. 33277388 PMC7962598

[36] Pérez MM, Pimentel VE, Fuzo CA, da Silva-Neto PV, Toro DM, Fraga-Silva TFC, Gardinassi LG, Oliveira CNS, Souza COS, Torre-Neto NT, de Carvalho JCS, De Leo TC, Nardini V, Feitosa MR, Parra RS, da Rocha JJR, Feres O, Vilar FC, Gaspar GG, Constant LF, Ostini FM, Degiovani AM, Amorim AP, Viana AL, Fernandes APM, Maruyama SR, Russo EMS, Santos IKFM, Bonato VLD, Cardoso CRB, Sorgi CA, Dias-Baruffi M, Faccioli LH. ImmunoCOVID Brazilian Research Consortium. 2022. Acetylcholine, fatty acids, and lipid mediators are linked to COVID-19 severity. J Immunol 209:250–261. doi:10.4049/jimmunol.2200079. 35768148

[37] Hamer M, Gale CR, Kivimäki M, Batty GD. 2020. Overweight, obesity, and risk of hospitalization for COVID-19: a community-based cohort study of adults in the United Kingdom. Proc Natl Acad Sci USA 117:21011–21013. doi:10.1073/pnas.2011086117. 32788355 PMC7474583

[38] Discacciati MG, Siani S, Campa A, Nakaya HI. 2022. Why should obese youth be prioritized in COVID-19 vaccination programs? A nationwide retrospective study. Lancet Reg Health Am 7:100167. doi:10.1016/j.lana.2021.100167. 35005691 PMC8720147

[39] Barberis E, Timo S, Amede E, Vanella VV, Puricelli C, Cappellano G, Raineri D, Cittone MG, Rizzi E, Pedrinelli AR, Vassia V, Casciaro FG, Priora S, Nerici I, Galbiati A, Hayden E, Falasca M, Vaschetto R, Sainaghi PP, Dianzani U, Rolla R, Chiocchetti A, Baldanzi G, Marengo E, Manfredi M. 2020. Large-scale plasma analysis revealed new mechanisms and molecules associated with the host response to SARS-CoV-2. Int J Mol Sci 21:E8623. doi:10.3390/ijms21228623. PMC7696386 33207699

[40] Oostdam A-V, Castañeda-Delgado JE, Oropeza-Valdez JJ, Borrego JC, Monárrez-Espino J, Zheng J, Mandal R, Zhang L, Soto-Guzmán E, Fernández-Ruiz JC, Ochoa-González F, Medinilla FMT, López JA, Wishart DS, Enciso-Moreno JA, López-Hernández Y. 2021. Immunometabolic signatures predict risk of progression to sepsis in COVID-19. PLoS One 16:e0256784. doi:10.1371/journal.pone.0256784. 34460840 PMC8405033

[41] Valdés A, Moreno LO, Rello SR, Orduña A, Bernardo D, Cifuentes A. 2022. Metabolomics study of COVID-19 patients in four different clinical stages. Sci Rep 12:1650. doi:10.1038/s41598-022-05667-0. 35102215 PMC8803913

[42] D’Alessandro A, Thomas T, Akpan IJ, Reisz JA, Cendali FI, Gamboni F, Nemkov T, Thangaraju K, Katneni U, Tanaka K, Kahn S, Wei AZ, Valk JE, Hudson KE, Roh D, Moriconi C, Zimring JC, Hod EA, Spitalnik SL, Buehler PW, Francis RO. 2021. Biological and clinical factors contributing to the metabolic heterogeneity of hospitalized patients with and without COVID-19. Cells 10:2293. doi:10.3390/cells10092293. 34571942 PMC8467961

[43] Song J-W, Lam SM, Fan X, Cao W-J, Wang S-Y, Tian H, Chua GH, Zhang C, Meng F-P, Xu Z, Fu J-L, Huang L, Xia P, Yang T, Zhang S, Li B, Jiang T-J, Wang R, Wang Z, Shi M, Zhang J-Y, Wang F-S, Shui G. 2020. Omics-driven systems interrogation of metabolic dysregulation in COVID-19 pathogenesis. Cell Metab 32:188–202.E5. doi:10.1016/j.cmet.2020.06.016. 32610096 PMC7311890

[44] Liu J, Li Z-B, Lu Q-Q, Yu Y, Zhang S-Q, Ke P-F, Zhang F, Li J-C. 2022. Metabolite profile of COVID-19 revealed by UPLC-MS/MS-based widely targeted metabolomics. Front Immunol 13:894170. doi:10.3389/fimmu.2022.894170. 35924246 PMC9339702

[45] Bellamine A, Pham TNQ, Jain J, Wilson J, Sahin K, Dallaire F, Seidah NG, Durkee S, Radošević K, Cohen ÉA. 2021. L-carnitine tartrate downregulates the ACE2 receptor and limits SARS-CoV-2 infection. Nutrients 13:1297. doi:10.3390/nu13041297. 33919991 PMC8071056

[46] Li C, Ou R, Wei Q, Shang H. 2021. Carnitine and COVID-19 susceptibility and severity: a Mendelian randomization study. Front Nutr 8:780205. doi:10.3389/fnut.2021.780205. 34901126 PMC8656944

[47] Páez-Franco JC, Torres-Ruiz J, Sosa-Hernández VA, Cervantes-Díaz R, Romero-Ramírez S, Pérez-Fragoso A, Meza-Sánchez DE, Germán-Acacio JM, Maravillas-Montero JL, Mejía-Domínguez NR, Ponce-de-León A, Ulloa-Aguirre A, Gómez-Martín D, Llorente L. 2021. Metabolomics analysis reveals a modified amino acid metabolism that correlates with altered oxygen homeostasis in COVID-19 patients. Sci Rep 11:6350. doi:10.1038/s41598-021-85788-0. 33737694 PMC7973513

[48] Gibellini L, De Biasi S, Paolini A, Borella R, Boraldi F, Mattioli M, Lo Tartaro D, Fidanza L, Caro-Maldonado A, Meschiari M, Iadisernia V, Bacca E, Riva G, Cicchetti L, Quaglino D, Guaraldi G, Busani S, Girardis M, Mussini C, Cossarizza A. 2020. Altered bioenergetics and mitochondrial dysfunction of monocytes in patients with COVID-19 pneumonia. EMBO Mol Med 12:e13001. doi:10.15252/emmm.202013001. 33078545 PMC7645870

[49] Bruzzone C, Bizkarguenaga M, Gil-Redondo R, Diercks T, Arana E, García de Vicuña A, Seco M, Bosch A, Palazón A, San Juan I, Laín A, Gil-Martínez J, Bernardo-Seisdedos G, Fernández-Ramos D, Lopitz-Otsoa F, Embade N, Lu S, Mato JM, Millet O. 2020. SARS-CoV-2 infection dysregulates the metabolomic and lipidomic profiles of serum. iScience 23:101645. doi:10.1016/j.isci.2020.101645. 33043283 PMC7534591

[50] Ghini V, Meoni G, Pelagatti L, Celli T, Veneziani F, Petrucci F, Vannucchi V, Bertini L, Luchinat C, Landini G, Turano P. 2022. Profiling metabolites and lipoproteins in COMETA, an Italian cohort of COVID-19 patients. PLoS Pathog 18:e1010443. doi:10.1371/journal.ppat.1010443. 35446921 PMC9022834

[51] Luporini RL, Pott-Junior H, Di Medeiros Leal MCB, Castro A, Ferreira AG, Cominetti MR, de Freitas Anibal F. 2021. Phenylalanine and COVID-19: tracking disease severity markers. Int Immunopharmacol 101:108313. doi:10.1016/j.intimp.2021.108313. 34741868 PMC8559801

[52] Shi D, Yan R, Lv L, Jiang H, Lu Y, Sheng J, Xie J, Wu W, Xia J, Xu K, Gu S, Chen Y, Huang C, Guo J, Du Y, Li L. 2021. The serum metabolome of COVID-19 patients is distinctive and predictive. Metabolism 118:154739. doi:10.1016/j.metabol.2021.154739. 33662365 PMC7920809

[53] Correia BSB, Ferreira VG, Piagge PMFD, Almeida MB, Assunção NA, Raimundo JRS, Fonseca FLA, Carrilho E, Cardoso DR. 2022. 1H qNMR-based metabolomics discrimination of Covid-19 severity. J Proteome Res 21:1640–1653. doi:10.1021/acs.jproteome.1c00977. 35674498

[54] Lawler NG, Gray N, Kimhofer T, Boughton B, Gay M, Yang R, Morillon A-C, Chin S-T, Ryan M, Begum S, Bong SH, Coudert JD, Edgar D, Raby E, Pettersson S, Richards T, Holmes E, Whiley L, Nicholson JK. 2021. Systemic perturbations in amine and kynurenine metabolism associated with acute SARS-CoV-2 infection and inflammatory cytokine responses. J Proteome Res 20:2796–2811. doi:10.1021/acs.jproteome.1c00052. 33724837

[55] Bartolini D, Stabile AM, Bastianelli S, Giustarini D, Pierucci S, Busti C, Vacca C, Gidari A, Francisci D, Castronari R, Mencacci A, Di Cristina M, Focaia R, Sabbatini S, Rende M, Gioiello A, Cruciani G, Rossi R, Galli F. 2021. SARS-CoV2 infection impairs the metabolism and redox function of cellular glutathione. Redox Biol 45:102041. doi:10.1016/j.redox.2021.102041. 34146958 PMC8190457

[56] Hall OJ, Klein SL. 2017. Progesterone-based compounds affect immune responses and susceptibility to infections at diverse mucosal sites. Mucosal Immunol 10:1097–1107. doi:10.1038/mi.2017.35. 28401937

[57] Mauvais-Jarvis F, Klein SL, Levin ER. 2020. Estradiol, progesterone, immunomodulation, and COVID-19 outcomes. Endocrinology 161:bqaa127. doi:10.1210/endocr/bqaa127. 32730568 PMC7438701

[58] Pinna G. 2021. Sex and COVID-19: a protective role for reproductive steroids. Trends Endocrinol Metab 32:3–6. doi:10.1016/j.tem.2020.11.004. 33229187 PMC7649655

[59] Lovre D, Bateman K, Sherman M, Fonseca VA, Lefante J, Mauvais-Jarvis F. 2021. Acute estradiol and progesterone therapy in hospitalised adults to reduce COVID-19 severity: a randomised control trial. BMJ Open 11:e053684. doi:10.1136/bmjopen-2021-053684. PMC8635865 34848523

[60] Zou Y, Kim CH, Chung JH, Kim JY, Chung SW, Kim MK, Im DS, Lee J, Yu BP, Chung HY. 2007. Upregulation of endothelial adhesion molecules by lysophosphatidylcholine. Involvement of G protein-coupled receptor GPR4. FEBS J 274:2573–2584. doi:10.1111/j.1742-4658.2007.05792.x. 17437524

[61] Huang YH, Schäfer-Elinder L, Wu R, Claesson H-E, Frostegård J. 1999. Lysophosphatidylcholine (LPC) induces proinflammatory cytokines by a platelet-activating factor (PAF) receptor-dependent mechanism. Clin Exp Immunol 116:326–331. doi:10.1046/j.1365-2249.1999.00871.x. 10337026 PMC1905274

[62] Theken KN, Tang SY, Sengupta S, FitzGerald GA. 2021. The roles of lipids in SARS-CoV-2 viral replication and the host immune response. J Lipid Res 62:100129. doi:10.1016/j.jlr.2021.100129. 34599996 PMC8480132

[63] Saccon E, Bandera A, Sciumè M, Mikaeloff F, Lashari AA, Aliberti S, Sachs MC, Billi F, Blasi F, Gabriel EE, Costantino G, De Roberto P, Krishnan S, Gori A, Peyvandi F, Scudeller L, Canetta C, Lorson CL, Valenti L, Singh K, Baldini L, Fracchiolla NS, Neogi U. COVID-19 Network Working Group. 2021. Distinct metabolic profile associated with a fatal outcome in COVID-19 patients during the early epidemic in Italy. Microbiol Spectr 9:e00549-21. doi:10.1128/Spectrum.00549-21. 34468185 PMC8565516

[64] Wong L-YR, Zheng J, Wilhelmsen K, Li K, Ortiz ME, Schnicker NJ, Thurman A, Pezzulo AA, Szachowicz PJ, Li P, Pan R, Klumpp K, Aswad F, Rebo J, Narumiya S, Murakami M, Zuniga S, Sola I, Enjuanes L, Meyerholz DK, Fortney K, McCray PB, Perlman S. 2022. Eicosanoid signalling blockade protects middle-aged mice from severe COVID-19. Nature 605:146–151. doi:10.1038/s41586-022-04630-3. 35314834 PMC9783543

[65] Zheng H, Jin S, Li T, Ying W, Ying B, Chen D, Ning J, Zheng C, Li Y, Li C, Chen C, Li X, Gao H. 2021. Metabolomics reveals sex-specific metabolic shifts and predicts the duration from positive to negative in non-severe COVID-19 patients during recovery process. Comput Struct Biotechnol J 19:1863–1873. doi:10.1016/j.csbj.2021.03.039. 33841749 PMC8021501

[66] Silwal P, Lim K, Heo J-Y, Park JI, Namgung U, Park S-K. 2018. Adenine attenuates lipopolysaccharide-induced inflammatory reactions. Korean J Physiol Pharmacol 22:379–389. doi:10.4196/kjpp.2018.22.4.379. 29962852 PMC6019877

[67] Fukuda T, Majumder K, Zhang H, Turner PV, Matsui T, Mine Y. 2016. Adenine inhibits TNF-α signaling in intestinal epithelial cells and reduces mucosal inflammation in a dextran sodium sulfate-induced colitis mouse model. J Agric Food Chem 64:4227–4234. doi:10.1021/acs.jafc.6b00665. 27166765

[68] Lee GY, Han SN. 2018. The role of vitamin E in immunity. Nutrients 10:1614. doi:10.3390/nu10111614. 30388871 PMC6266234

[69] Ahsan H, Ahad A, Iqbal J, Siddiqui WA. 2014. Pharmacological potential of tocotrienols: a review. Nutr Metab (Lond) 11:52. doi:10.1186/1743-7075-11-52. 25435896 PMC4247006

[70] Asbaghi O, Sadeghian M, Nazarian B, Sarreshtedari M, Mozaffari-Khosravi H, Maleki V, Alizadeh M, Shokri A, Sadeghi O. 2020. The effect of vitamin E supplementation on selected inflammatory biomarkers in adults: a systematic review and meta-analysis of randomized clinical trials. Sci Rep 10:17234. doi:10.1038/s41598-020-73741-6. 33057114 PMC7560744

[71] Kelley KW, Kent S. 2020. The legacy of sickness behaviors. Front Psychiatry 11:607269. doi:10.3389/fpsyt.2020.607269. 33343432 PMC7744348

[72] Usmani OS, Belvisi MG, Patel HJ, Crispino N, Birrell MA, Korbonits M, Korbonits D, Barnes PJ. 2005. Theobromine inhibits sensory nerve activation and cough. FASEB J 19:231–233. doi:10.1096/fj.04-1990fje. 15548587

[73] National Institutes of Health. 2020. COVID-19 treatment guidelines. https://www.covid19treatmentguidelines.nih.gov/. 34003615

[74] World Health Organization. 2020. Clinical management of COVID-19: interim guidance (WHO). https://www.paho.org/en/documents/clinical-management-covid-19-interim-guidance-who.

[75] Yu T, Park Y, Johnson JM, Jones DP. 2009. apLCMS—adaptive processing of high-resolution LC/MS data. Bioinformatics 25:1930–1936. doi:10.1093/bioinformatics/btp291. 19414529 PMC2712336

[76] Domingo-Almenara X, Guijas C, Billings E, Montenegro-Burke JR, Uritboonthai W, Aisporna AE, Chen E, Benton HP, Siuzdak G. 2019. The METLIN small molecule dataset for machine learning-based retention time prediction. Nat Commun 10:5811. doi:10.1038/s41467-019-13680-7. 31862874 PMC6925099

[77] Huan T, Tang C, Li R, Shi Y, Lin G, Li L. 2015. MyCompoundID MS/MS search: metabolite identification using a library of predicted fragment-ion-spectra of 383,830 possible human metabolites. Anal Chem 87:10619–10626. doi:10.1021/acs.analchem.5b03126. 26415007

