# Supplementary material for: Integrated Metabolic and Inflammatory Signatures Associated with Severity of, Fatality of, and Recovery from COVID-19
Source: Microbiol Spectr. 2023 Feb 28;11(2):e02194-22. doi: 10.1128/spectrum.02194-22 (PMC10100880; doi:10.1128/spectrum.02194-22)
Supplement: Supplemental file 1 — Tables S1 and S2, Fig. S1 to S7, and Data S2. Download spectrum.02194-22-s0001.pdf, PDF file, 1.0 MB [file spectrum.02194-22-s0001.pdf]

Table S1. Clinical, demographic, and laboratorial data from the study population

	Control N=27	Mild N=28	Moderate N=40	Severe N=49	Fatal N=33	p value
Demographic characteristics						
Age, median (IQR)	56 (50-60)	31 (23-49)	58 (45-74)	57 (46-69)	65 (59-71)	a, e, f, g
Sex. No (%)						
Male	9 (33)	11 (39)	22 (55)	27 (55)	22 (67)	d, g
Female	18 (67)	17 (61)	18 (45)	22 (45)	11 (33)	
Days post symptom, median (IQR)	-	8 (6-10)	12 (9-18)	11 (9-15)	17 (10-25)	e, f, g, j
Comorbidities and risk factors. No (%)						
Obesity	6 (22.2)	-	4 (10.0)	14 (28.6)	5 (15.1)	h
Hypertension	7 (29.2)	3 (10.7)	15 (37.5)	20 (40.8)	17 (51.5)	e, f, g
Diabetes	1 (3.7)	2 (7.1)	11 (27.5)	13 (26.5)	12 (36.3)	b, c, d, g
Dyslipidemia	1 (3.7)	-	3 (7.5)	4 (8.2)	3 (9.1)	ns
Heart disease	3 (11.1)	1 (3.6)	9 (22.5)	9(18.4)	9 (27.2)	e, g
Chronic Renal Disease	-	-	4 (10.0)	1 (2.0)	5 (15.1)	j
Chronic Obstructive Pulmonary Disease	1 (3.7)	-	-	1 (2.0)	5 (15.1)	j
Pregnancy	-	3 (17.4)	2 (11.1)	3 (13.6)	-	ns
Symptoms. No (%)						
Fever	-	12 (42.8)	19 (47.5)	23 (46.9)	25 (75.7)	g, i, j
Cough	-	14 (50.0)	25 (62.5)	34 (69.4)	21 (63.6)	ns
Dyspnea	-	9 (32.1)	17 (42.5)	36 (73.5)	23 (69.7)	f, g, h, i
Myalgia/Arthralgia	-	10 (35.7)	11 (27.5)	22 (45.0)	9 (27.3)	ns
Anosmia	-	5 (17.8)	4 (10.0)	2 (4.1)	1 (3.0)	ns
Chest pain	-	3 (10.7)	-	4 (8.2)	3 (9.0)	ns
Diarrhea	-	8 (28.6)	3 (7.5)	11 (22.4)	3 (9.0)	e
Headache	-	15 (53.6)	11 (27.5)	13 (26.5)	9 (27.2)	e, f
Abdominal pain	-	4 (14.3)	3 (7.5)	5 (10.2)	4 (12.1)	ns
Vomiting	-	5 (17.8)	4 (10.0)	8 (16.3)	6 (18.1)	ns

	Control N=27	Mild N=28	Moderate N=40	Severe N=49	Fatal N=33	p value
Asthenia	-	8 (28.6)	-	16 (32.7)	13 (39.4)	ns
Blood routine						
Erythrocytes (10^6/mm^3)	4.8 (4.5-5.1)	4.6 (4.3-5.3)	4.4 (3.8-4.9)	4.3 (3.8-4.9)	3.2 (2.8-4.3)	d, g, h, i, j
Hemoglobin (g/dL)	14.3 (13.5-14.8)	14.5 (12.3-15.7)	12.7 (11.0-14.4)	12.8 (11.3-14.0)	9.5 (8.4-13.0)	d, h, i, j
Leukocyte counts (/mm^3)	6.7 (5.4-7.5)	7.5 (5.5-9.2)	8.3 (6.3-11.9)	7.7 (4.7-10.4)	12.1 (8.9-15.5)	d, g, j
Neutrophil counts (/mm^3)	3.9 (2.9-4.6)	4.8 (2.5-6.4)	5.8 (4.1-8.0)	5.6 (3.5-8.5)	8.7 (6.7-11.8)	b, c, d, g, i, j
Lymphocyte counts (/mm^3)	2.2 (1.6-2.8)	2.1 (1.7-2.5)	1.7 (1.0-2.2)	1.3 (1.0-1.8)	0.7 (0.5-1.3)	c, d, g, h, i, j
Monocyte counts (/mm^3)	339.0 (189.0-553.5)	275.0 (226.0-431.0)	411.0 (271.3-738.5)	425.0 (250.5-662.0)	447.0 (282.5-731.5)	ns
Platelet counts (/mm^3)	231.5 (195.3-285.8)	240.0 (182.4-277.4)	227.8 (180.2-298.5)	214.6 (180.3-317.9)	173.3 (108.2-227.7)	i, j
Coagulation function, mean ± SD						
Activated partial thromboplastin time (s)	-	30.0 (28.8-31.4)	30.0 (29.0-32.2)	30.0 (29.0-33.2)	31.5 (27.4-37.2)	g
Prothrombin time (s)	-	13.0 (12.0-13.2)	13.5 (12.6-14.1)	13.5 (12.2-14.5)	14.1 (13.0-15.7)	ns
D-dimer (mg/dL)	-	306.6 (218.3-611.3)	374.0 (72.2-764.5)	423.0 (2.9-1004.0)	571 (262.8-1113.0)	ns
Blood biochemistry, mean ± SD						
Albumin (mg/dL)	3.5 – 4.8	4.3 (4.2-4.4)	3.0 (2.7-3.5)	3.3 (3.0-3.7)	3.2 (2.8-3.5)	2.6 (2.3-3.2)
Alanine aminotransferase (UI/L)	<55	18 (14-26)	25 (10-56)	31 (17-56)	41 (25-62)	43 (24-67)
Aspartate aminotransferase (UI/L)	5 – 34	22 (19-27)	23 (13-33)	32 (18-51)	41 (27-69)	40 (30-63)
Total bilirubin (mg/dL)	≤1.2	0.6 (0.4-0.8)	0.3 (0.2-0.4)	0.4 (0.3-0.6)	0.5 (0.3-0.6)	0.5 (0.3-0.8)
Creatinine (mg/dL)	0.6 – 1.3	0.8 (0.7-1.0)	0.7 (0.5-0.9)	0.9 (0.8-1.2)	0.9 (0.7-1.1)	1.5 (1.0-2.8)
C-reactive protein (mg/dL)	<0.5	0.2 (0.1-0.6)	3.2 (2.1-8.1)	8.4 (2.3-29.1)	9.2 (2.3-42.2)	12.1 (8.0-22.9)
Ferritin (ng/mL)	21.8 – 274.6	150.0 (94.1-208.0)	364.1 (209.4-760.5)	526.2 (275.6-1295.0)	741.1 (299.5-1186.0)	1180.0 (732.7-2642.0)
Hospital support. No. (%)						
Infirmary	-	6 (21.4)	40 (100.0)	24 (49.0)	-	e, f, h
ICU	-	-	-	25 (51.0)	33 (100.0)	j

	Control N=27	Mild N=28	Moderate N=40	Severe N=49	Fatal N=33	p value
Respiratory support received. No. (%)						
Nasal Catheter	-	4 (14.3)	40 (100.0)	38 (77.6)	14 (42.4)	e, f, h
Non-rebreathing mask	-	-	-	4 (8.2)	3 (9.0)	ns
Invasive mechanical ventilation	-	-	-	11 (22.4)	27 (81.8)	j
Pharmacological Medications. No. (%)						
Azithromycin	-	7 (25.0)	18 (45.0)	13 (26.5)	14 (42.4)	
Ceftriaxone	-	2 (7.1)	18 (45.0)	17 (34.7)	19 (57.5)	
Dexamethasone	-	2 (7.1)	10 (25.0)	15 (30.6)	16 (48.4)	
Enoxaparin	-	1 (3.6)	12 (30.0)	13 (26.5)	-	

Abbreviations: COPC, chronic obstructive pulmonary disease; No., number or values; ICU, Intensive Care Unit; s, seconds; IQR, Interquartile range; ns, not significant. Categorical variables represented as number (percentage) were evaluated using Fisher's exact test. Continuous variables represented as median (interquartile range) were evaluated with one-way analysis of variance (ANOVA) and Kruskal-Walli's test. Significant differences were considered when p <0.05. Significance is given as: a = control *versus* mild; b = control *versus* moderate; c = control *versus* severe; d = control *versus* fatal; e = mild *versus* moderate; f = mild *versus* severe, g = mild *versus* fatal; h = moderate *versus* severe; i = moderate *versus* fatal; j = severe *versus* fatal.

Table S2. Clinical, demographic, and laboratorial data of individuals recovering from acute COVID-19

	Recovery N=20
Demographic characteristics	
Age mean ± SD	56 ± 15.2
Sex, No (%)	
Male	9 (45.0)
Female	11 (55.0)
Days post symptom onset collection ± SD	
Mild (n=4)	167 ± 23
Moderate (n=6)	171 ± 7
Severe (n=10)	188 ± 44
Comorbidities, No (%)	
Obesity	2 (10.0)
Hypertension	7 (35.0)
Diabetes *mellitus*	4 (20.0)
Dyslipidemia	3 (15.0)
Heart disease	5 (25.0)
Chronic Kidney Disease	2 (10.0)
Blood routine*	
Erythrocytes, $\times 10^6/mm^3$	4.54 ± 0.7
Hemoglobin (g/dL)	13.1 ± 2.1
Leucocytes count ($/mm^3$)	6.8 ± 1.8
Neutrophils count ($/mm^3$)	3.3 ± 1.8
Lymphocytes count ($/mm^3$)	2.5 ± 1.1
Monocytes count ($/mm^3$)	262 ± 159
Platelets count ($/mm^3$)	238 ± 110
Symptoms post covid-19, No (%)	
Anosmia/ ageusia	3 (21.4)
Leg pain	2 (14.3)
Shortness of breath	4 (28.6)
Headache	1 (7.1)
Abdominal pain	3 (21.4)
Back pain	5 (35.7)
Memory loss	2 (14.3)
Fatigue	5 (35.7)
Inappetence	3 (21.4)
Worsening of vision	4 (28.6)
Asymptomatic	6 (30.0)

*All parameters measured on blood routine are within normal ranges

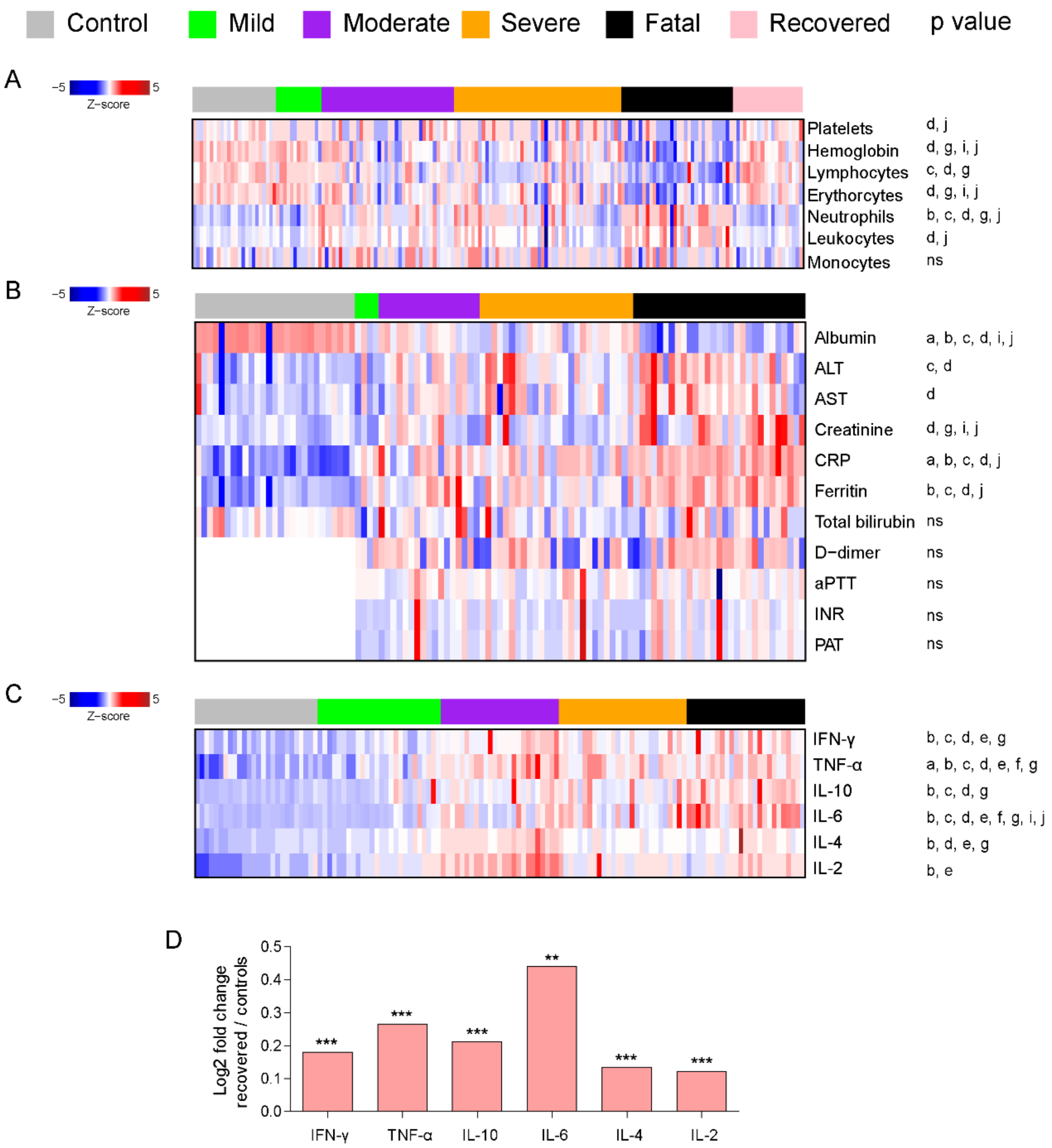

Fig. S1 – Hematological, biochemical and cytokine measurements. (A) Hierarchical clustering of hematological parameters. (B) Hierarchical clustering of acute phase/coagulation parameters. (C) Cytokine measurements compared between individuals with COVID-19 and control donors. The color scale from blue to red reflects lower to higher abundance, respectively. D-dimer, PAT, aPTT and INR were not measured for control samples. Statistical analyses were performed with one-way analysis of variance (ANOVA) and Tukey's multi comparison test. Significance is given as: a = control *versus* mild; b = control *versus* moderate; c = control *versus* severe; d = control *versus* fatal; e = mild *versus* moderate; f = mild *versus* severe; g = mild *versus* fatal, h = moderate *versus* severe; i = moderate *versus* fatal; j = severe *versus* fatal; k = control *versus* recovery evaluated with T-test. Differences were considered significant when $p < 0.05$. (D) Log2 fold change of cytokine measurements comparing individuals recovered from acute COVID-19 and control donors, evaluated with T-test. ** $p < 0.01$, *** $p < 0.001$. Abbreviations: aPTT – activated partial thromboplastin time; PAT – prothrombin time; AST – aspartate aminotransferase; ALT – alanine aminotransferase; CRP – C reactive protein; INR – International normalized ratio.

	Age (1314)	Sex (1193)	Heart_disease (733)	Hypertension (1330)	Diabetes (734)	Dyslipidemia (448)	Obesity (550)	CRD (1304)	COPD (338)
Infection (1003)	474	286	83	231	118	37	39	211	40
Age (1314)		254	103	283	176	46	89	185	50
Sex (1193)			90	176	99	51	61	243	56
Heart_disease (733)				197	68	41	48	99	36
Hypertension (1330)					101	48	68	157	70
Diabetes (734)						40	64	98	23
Dyslipidemia (448)							19	65	17
Obesity (550)								92	17
CRD (1304)									87

Fig. S2 – Overlap between significant metabolite features associated with SARS-CoV-2 infection, age, sex, heart disease, hypertension, diabetes, dyslipidemia, obesity, chronic renal disease (CRD) and chronic obstructive pulmonary disease (COPD). Each number in parentheses represents the total number of features associated with each variable. Each number reflects on the columns represent the overlap between two variables. E.g. Infection (1003) and Age (1314) share 474 significant metabolite features.

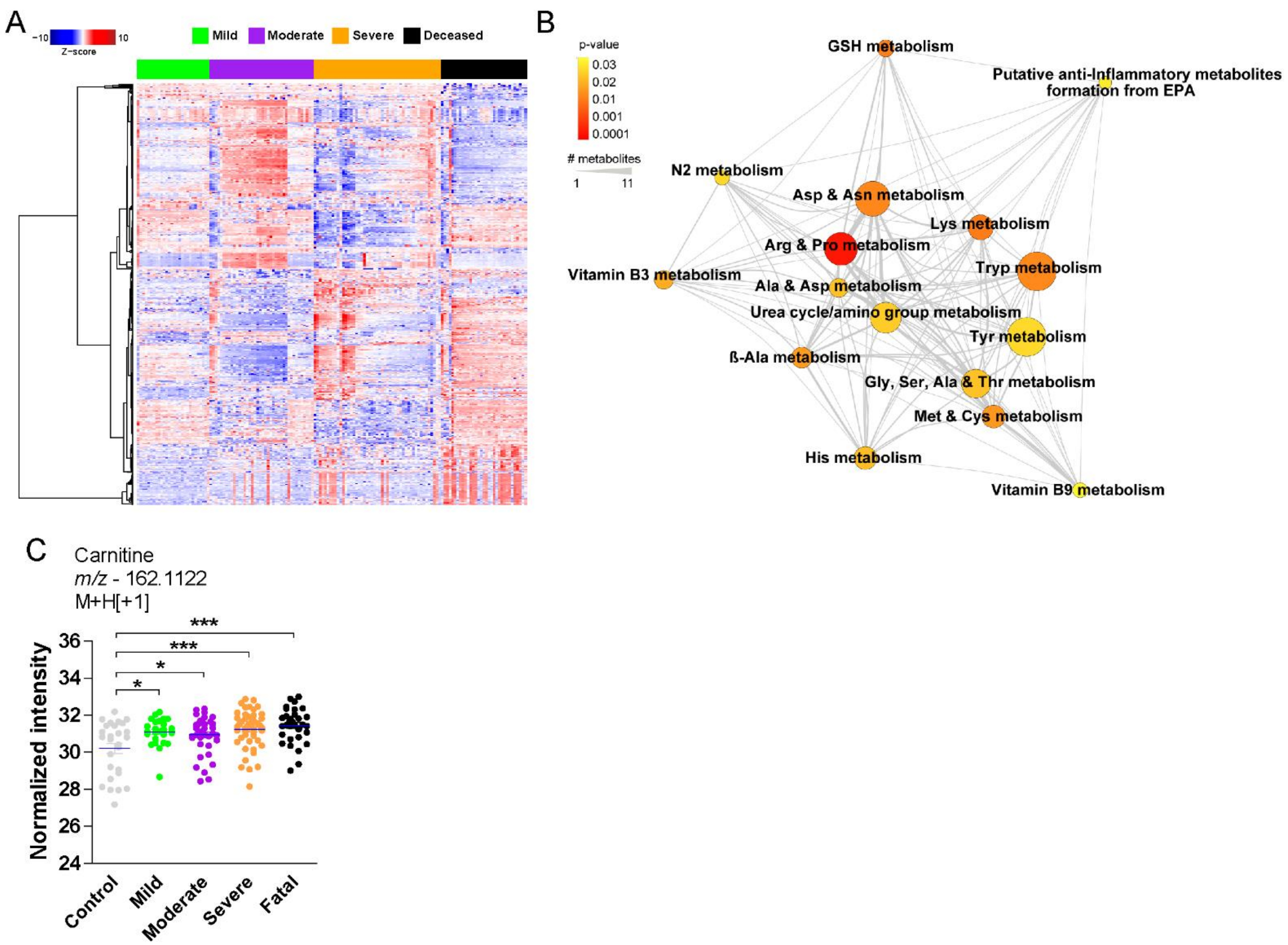

Fig. S3 – Differential metabolite features between categories of COVID-19 severity. (A) One-way hierarchical clustering of significant metabolite features resulting from analysis of variance (ANOVA) between categories of COVID-19 severity. (B) Mummichog pathway analysis of significant metabolite features in A. The size of nodes in the network depicts the number of significant metabolites in each pathway and the color scale represents the significance. Nodes were linked by the number of metabolite features shared between each pathway. (C) Abundance of carnitine compared between categories of severity. Significance levels are shown as * $p < 0.05$, *** $p < 0.001$.

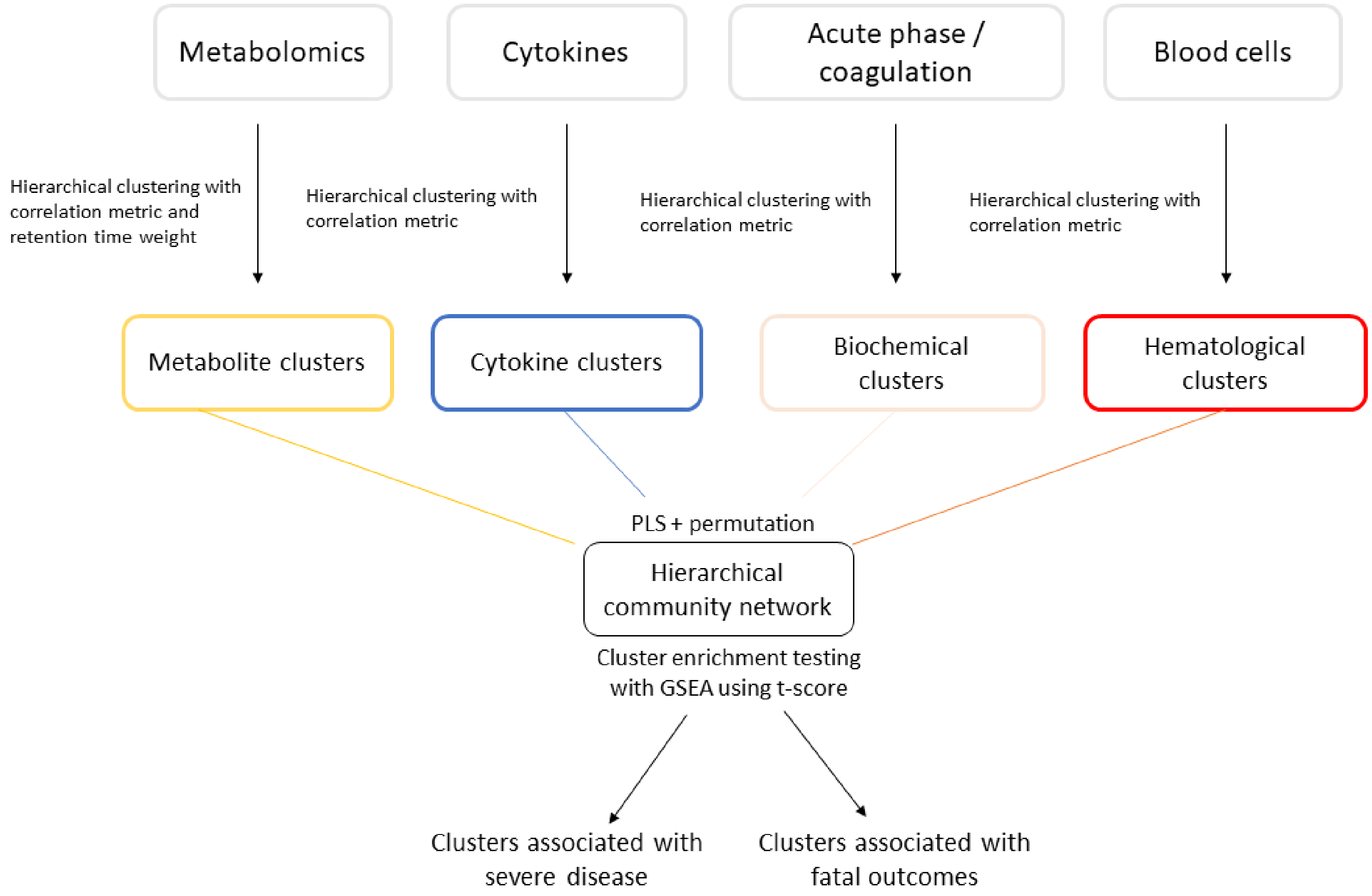

Fig. S4 – Hierarchical community network approach. Clusters of metabolites, cytokines, acute phase / coagulation, and hematological features were assessed via hierarchical clustering using correlation metric as distance. Enforcing similar chromatographic retention time among metabolites groups features from to the same chemical class or pathway. Clusters from different data types were then used as input in partial least square (PLS) regression to determine associations. Significance was assessed via permutation for each pair of data. The leading network was then queried with gene set enrichment analysis to identify clusters that predict disease severity and fatal outcomes of COVID-19. See more details on the methods section.

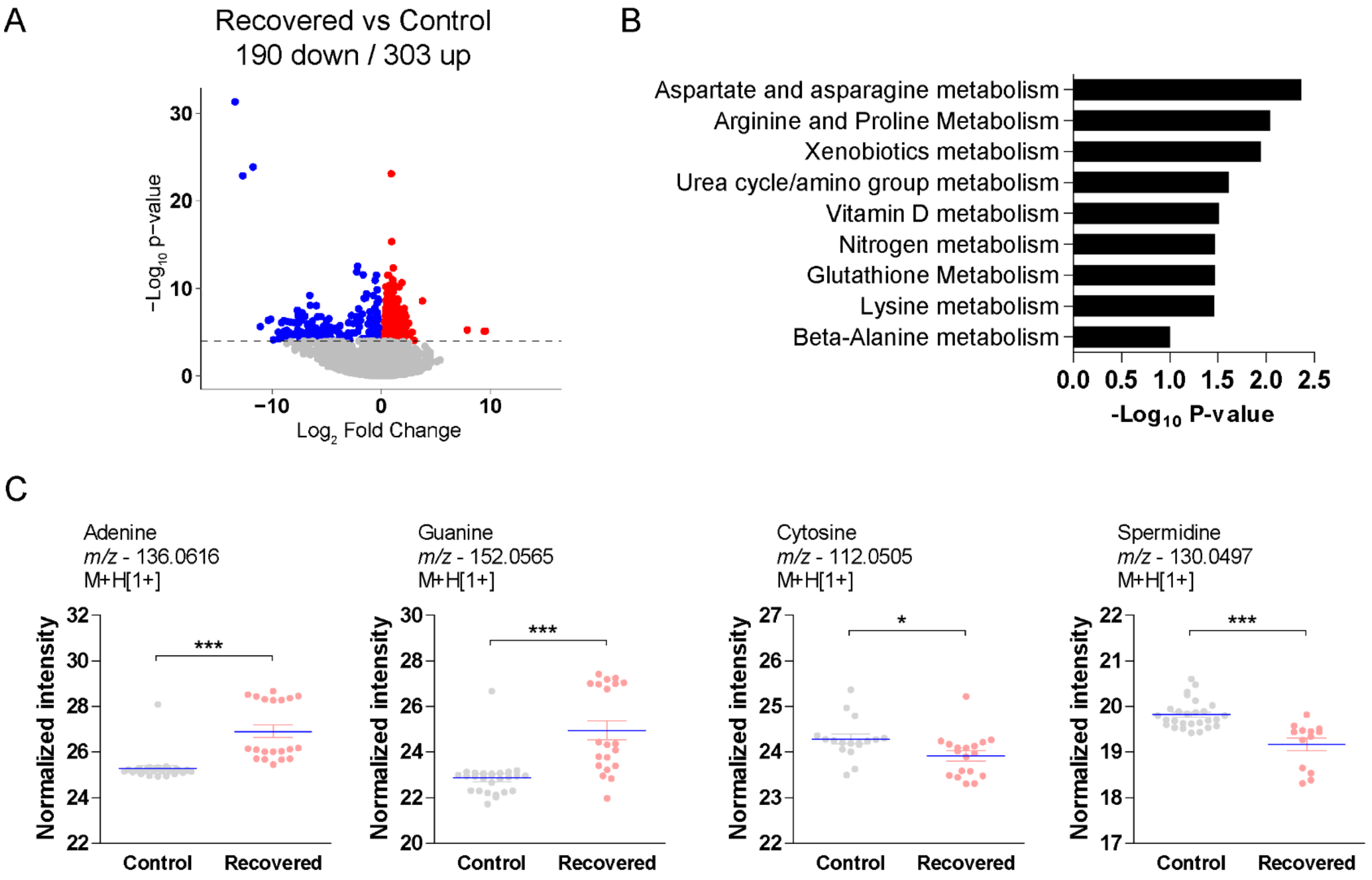

Fig. S5 – Differential metabolite features between patients recovered from COVID-19 and controls. (A) Volcano plot showing significant metabolite features in plasma of patients recovered from acute COVID-19 compared to controls. (B) Mummichog pathway analysis of significant metabolite features. (C) Differential abundance of adenine, guanine, cytosine, and spermidine. Significance levels are shown as * $p < 0.05$, *** $p < 0.001$.

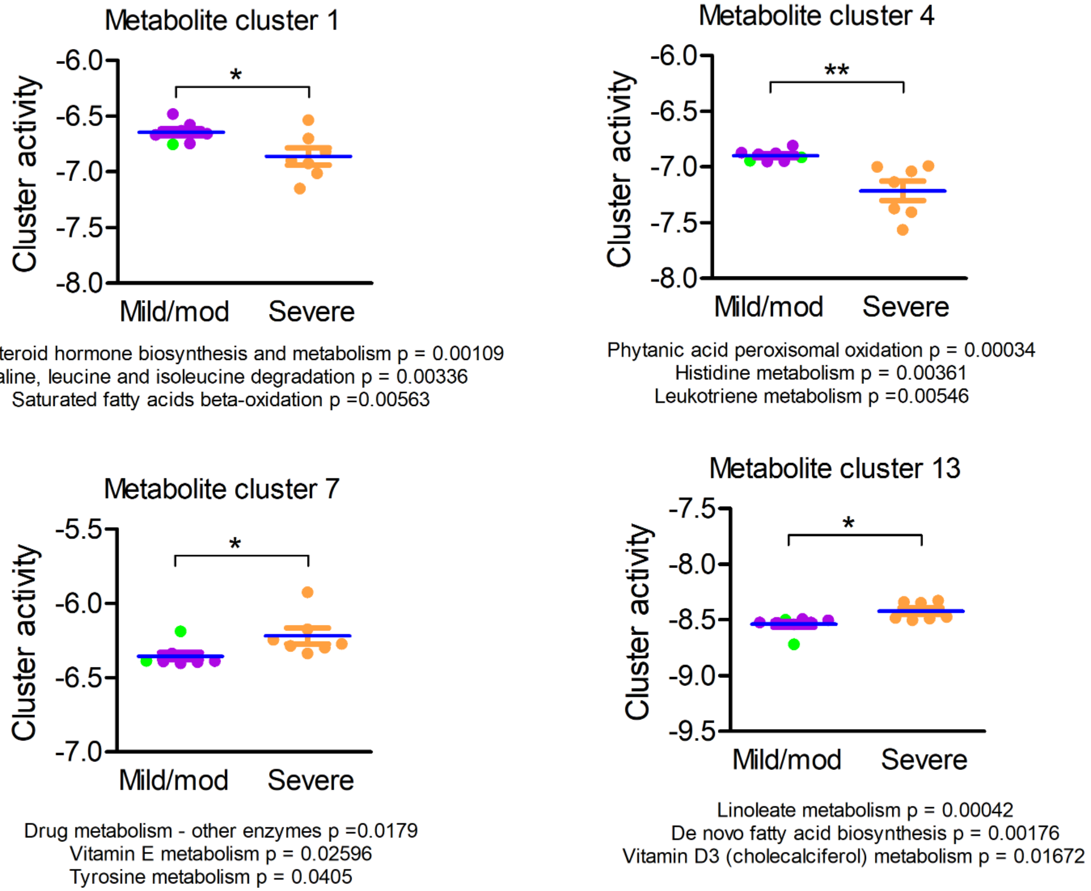

Fig. S6 – Differential clusters activity between individuals recovered from mild-to-moderate and severe COVID-19. The activity of metabolite clusters 1, 4, 7 and 13 was compared between individuals recovered from mild together with moderate disease with individuals recovering from severe disease. Mummichog software was used to predict enriched metabolic pathways and the 3 most significant are shown for each cluster. Significance levels are shown as * $p < 0.05$, ** $p < 0.01$.

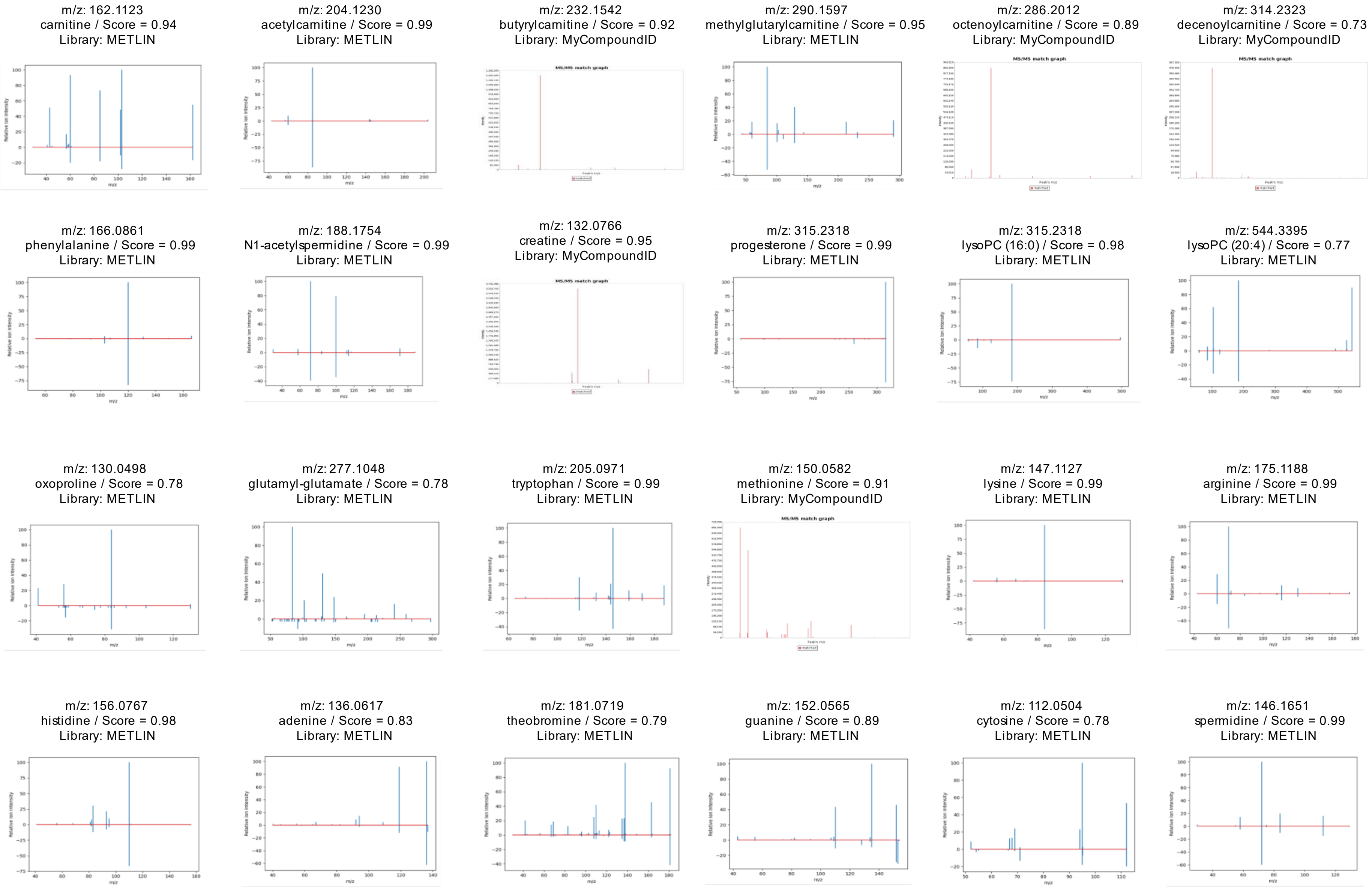

Fig. S7 – Metabolite annotation via fragment similarity matching to reference spectral libraries. Features matching compounds at MS1 accurate mass and MS2 fragments with a score > 0.7 were annotated at level 2 of identification according to the Metabolomics Standard Initiative. Mirror plots generated with METLIN Gen 2 (https://metlincloud2.massconsortium.com/) show input data on the bottom and library data at the top. Plots generated with MyCompoundID show matching peaks from input data in red.

Data S1 – Phenotypic data and annotations generated with mummichog software (.xlsx file)

Data S2 – Code used for moderated T-test, moderated F-test, and multivariate linear and logistic regressions.

```
#### From supplemental material ###
# Data S1 - Save the phenotypic data as "Pheno_data.txt"

#### From the Metabolomics Workbench database – identifier ST002291####
# Download the feature table in the results file
# Format the feature table to include only the columns and lines of m/z, retention time and intensity values as below

# Filter, transform and normalize the feature table as described in the material and methods
# Save the feature table as "Feature_table.txt"

#### input and format data #####
data <- read.delim( file = "Feature_table.txt"  , header = T)
mz_rt <- data[,1:2]
mz_rt <- paste(mz_rt[,1], mz_rt[,2], sep="_")
data_matrix <- as.matrix(data[,-1:-2])
row.names(data_matrix) <- mz_rt

pheno_data <- read.delim("Pheno_data.txt", header = T)


#### compute differentially abundant metabolite features (DAM) using moderated T-test according to infection status #######
library(limma)

design <- model.matrix(~0+Infection_status, data = pheno_data)
colnames(design) <- c("neg", "pos", "rec")
fit <- lmFit(data_matrix, design = design)
contrast.matrix <- makeContrasts(pos-neg, levels = design)
fit2 <- contrasts.fit(fit, contrast.matrix)
fit2 <- eBayes(fit2)
DAM_infection <- topTable(fit2, number=Inf, adjust.method = "fdr")

#### compute DAM adjusting for confounding variables, according to category of severity compared to controls #######
```

```
library(limma)

design1 <- model.matrix(~0+Class1+Sex+Age+Heart_disease+Hypertension+Diabetes+Dyslipidemia+
                         Obesity+CRD+COPD, data = pheno_data)
colnames(design1) <- c("ctrl", "fatal", "mild","moder","recov","sever","sex","age","heart_disease",
                       "hypertension","diabetes","dyslipidemia","obesity","CRD","COPD")
fit <- lmFit(data_matrix, design = design1)

### for mild disease####
contrast.matrix_mild <- makeContrasts(mild-ctrl, levels = design1)
fit2 <- contrasts.fit(fit, contrast.matrix_mild)
fit2 <- eBayes(fit2)
DAM_mild <- topTable(fit2, number=Inf, adjust.method = "fdr")

### for moderate disease####
contrast.matrix_moder <- makeContrasts(moder-ctrl, levels = design1)
fit2 <- contrasts.fit(fit, contrast.matrix_moder)
fit2 <- eBayes(fit2)
DAM_moder <- topTable(fit2, number=Inf, adjust.method = "fdr")

### for severe disease####
contrast.matrix_sever <- makeContrasts(sever-ctrl, levels = design1)
fit2 <- contrasts.fit(fit, contrast.matrix_sever)
fit2 <- eBayes(fit2)
DAM_sever <- topTable(fit2, number=Inf, adjust.method = "fdr")

### for fatal disease####
contrast.matrix_fatal <- makeContrasts(fatal-ctrl, levels = design1)
fit2 <- contrasts.fit(fit, contrast.matrix_fatal)
fit2 <- eBayes(fit2)
DAM_fatal <- topTable(fit2, number=Inf, adjust.method = "fdr")

### for recovery after acute disease ####
contrast.matrix_recov <- makeContrasts(recov-ctrl, levels = design1)
fit2 <- contrasts.fit(fit, contrast.matrix_recov)
fit2 <- eBayes(fit2)
DAM_recov <- topTable(fit2, number=Inf, adjust.method = "fdr")
```

```
#### compute DAM between groups of individuals with COVID-19 using moderated F-test ####
data_matrix2 <- data_matrix[,-129:-148]
data_matrix2 <- data_matrix2[,-1:-27]

pheno_data2 <- pheno_data[-129:-148,]
pheno_data2 <- pheno_data2[-1:-27,]

design2 <- model.matrix(~0+Class1+Sex+Age+Heart_disease+Hypertension+Diabetes+Dyslipidemia+
                         Obesity+CRD+COPD, data = pheno_data2)
colnames(design2) <- c("fatal", "mild","moder","sever","sex","age","heart_disease",
                       "hypertension","diabetes","dyslipidemia","obesity","CRD","COPD")
fit <- lmFit(data_matrix2, design = design2)

contrast.matrix_anova <- makeContrasts(mild-moder,mild-sever, mild-fatal,
                                       moder-sever, moder-fatal,
                                       sever-fatal, levels = design2)
fit2 <- contrasts.fit(fit, contrast.matrix_anova)
fit2 <- eBayes(fit2)
DAM_anova <- topTable(fit2, number=Inf, adjust.method = "fdr")


#### compute metabolite features associated with SpO2 via linear regression ####
library(epicalc)
library(broom)

data_matrix3 <- data_matrix[,-129:-148]
pheno_data3 <- pheno_data[-129:-148,]

plist <- c(1:9893)
betalist <- c(1:9893)
statistic <- c(1:9893)

for (k in 1:9893) {
  model1 <- glm(unlist(pheno_data$SpO2) ~ unlist(data_matrix[k, 1:197]) + pheno_data$Age + pheno_data$Sex +
                pheno_data$Heart_disease + pheno_data$Hypertension + pheno_data$Diabetes +
                pheno_data$Dyslipidemia + pheno_data$Obesity + pheno_data$CRD + pheno_data$COPD)
```

```
  model0 <- glm(unlist(pheno_data$SpO2) ~  pheno_data$Age + pheno_data$Sex +
                pheno_data$Heart_disease + pheno_data$Hypertension + pheno_data$Diabetes +
                pheno_data$Dyslipidemia + pheno_data$Obesity + pheno_data$CRD + pheno_data$COPD)
  test.m <- lrtest( model1, model0 )
  statistic[k] <- tidy(model1)$statistic[2]
  plist[k] <- test.m$p.value
  betalist[k] <- coef(model1)[2] }
p_adjust <- data.frame(p.adjust(as.matrix(plist), method ="fdr"))
Spo2_associat <- cbind(mz_rt,betalist, statistic, plist, p_adjust)


#### compute metabolite features associated with co-variables with linear or logistic regression ####
library(epicalc)
library(broom)

data_matrix3 <- data_matrix[,-129:-148]
pheno_data3 <- pheno_data[-129:-148,]

##### age ######

plist <- c(1:9893)
betalist <- c(1:9893)
statistic <- c(1:9893)

for (k in 1:9893) {
  model1 <- glm(pheno_data3$Age  ~  unlist(data_matrix3[k, 1:177]) + pheno_data3$Sex + pheno_data3$Heart_disease
              + pheno_data3$Hypertension + pheno_data3$Diabetes + pheno_data3$Dyslipidemia + pheno_data3$Obesity
              + pheno_data3$CRD + pheno_data3$COPD)
  model0 <- glm(pheno_data3$Age  ~   pheno_data3$Sex + pheno_data3$Heart_disease
              + pheno_data3$Hypertension + pheno_data3$Diabetes + pheno_data3$Dyslipidemia + pheno_data3$Obesity
              + pheno_data3$CRD + pheno_data3$COPD)
  test.m <- lrtest( model1, model0 )
  statistic[k] <- tidy(model1)$statistic[2]
  plist[k] <- test.m$p.value
  betalist[k] <- coef(model1)[2] }
```

```
p_adjust <- p.adjust(as.matrix(plist), method ="fdr")

Age_associat <- cbind(mz_rt,betalist, statistic, plist, p_adjust)


##### sex ######

plist <- c(1:9893)
betalist <- c(1:9893)
statistic <- c(1:9893)

for (k in 1:9893) {
  model1 <- glm(pheno_data3$Sex  ~  unlist(data_matrix3[k, 1:177]) + pheno_data3$Age + pheno_data3$Heart_disease
                + pheno_data3$Hypertension + pheno_data3$Diabetes + pheno_data3$Dyslipidemia + pheno_data3$Obesity
                + pheno_data3$CRD + pheno_data3$COPD, family = binomial)
  model0 <- glm(pheno_data3$Sex  ~   pheno_data3$Age + pheno_data3$Heart_disease
                + pheno_data3$Hypertension + pheno_data3$Diabetes + pheno_data3$Dyslipidemia + pheno_data3$Obesity
                + pheno_data3$CRD + pheno_data3$COPD, family = binomial)
  test.m <- lrtest( model1, model0 )
  statistic[k] <- tidy(model1)$statistic[2]
  plist[k] <- test.m$p.value
  betalist[k] <- coef(model1)[2] }

p_adjust <- p.adjust(as.matrix(plist), method ="fdr")

Sex_associat <- cbind(mz_rt,betalist, statistic, plist, p_adjust)

##### heart_disease ######

plist <- c(1:9893)
betalist <- c(1:9893)
statistic <- c(1:9893)

for (k in 1:9893) {
  model1 <- glm(pheno_data3$Heart_disease  ~  unlist(data_matrix3[k, 1:177]) + pheno_data3$Sex + pheno_data3$Age
                + pheno_data3$Hypertension + pheno_data3$Diabetes + pheno_data3$Dyslipidemia + pheno_data3$Obesity
                + pheno_data3$CRD + pheno_data3$COPD, family = binomial)
```

```
  model0 <- glm(pheno_data3$Heart_disease  ~   pheno_data3$Sex + pheno_data3$Age
               + pheno_data3$Hypertension + pheno_data3$Diabetes + pheno_data3$Dyslipidemia + pheno_data3$Obesity
               + pheno_data3$CRD + pheno_data3$COPD, family = binomial)
  test.m <- lrtest( model1, model0 )
  statistic[k] <- tidy(model1)$statistic[2]
  plist[k] <- test.m$p.value
  betalist[k] <- coef(model1)[2] }

p_adjust <- p.adjust(as.matrix(plist), method ="fdr")

Heart_disease_associat <- cbind(mz_rt,betalist, statistic, plist, p_adjust)

##### hypertension ######

plist <- c(1:9893)
betalist <- c(1:9893)
statistic <- c(1:9893)

for (k in 1:9893) {
  model1 <- glm(pheno_data3$Hypertension  ~  unlist(data_matrix3[k, 1:177]) + pheno_data3$Sex + pheno_data3$Heart_disease
               + pheno_data3$Age + pheno_data3$Diabetes + pheno_data3$Dyslipidemia + pheno_data3$Obesity
               + pheno_data3$CRD + pheno_data3$COPD, family = binomial)
  model0 <- glm(pheno_data3$Hypertension  ~   pheno_data3$Sex + pheno_data3$Heart_disease
               + pheno_data3$Age + pheno_data3$Diabetes + pheno_data3$Dyslipidemia + pheno_data3$Obesity
               + pheno_data3$CRD + pheno_data3$COPD, family = binomial)
  test.m <- lrtest( model1, model0 )
  statistic[k] <- tidy(model1)$statistic[2]
  plist[k] <- test.m$p.value
  betalist[k] <- coef(model1)[2] }

p_adjust <- p.adjust(as.matrix(plist), method ="fdr")

Hypertension_associat <- cbind(mz_rt,betalist, statistic, plist, p_adjust)

##### diabetes ######

plist <- c(1:9893)
```

```
betalist <- c(1:9893)
statistic <- c(1:9893)

for (k in 1:9893) {
  model1 <- glm(pheno_data3$Diabetes  ~  unlist(data_matrix3[k, 1:177]) + pheno_data3$Sex + pheno_data3$Heart_disease
                + pheno_data3$Hypertension + pheno_data3$Age + pheno_data3$Dyslipidemia + pheno_data3$Obesity
                + pheno_data3$CRD + pheno_data3$COPD, family = binomial)
  model0 <- glm(pheno_data3$Diabetes  ~   pheno_data3$Sex + pheno_data3$Heart_disease
                + pheno_data3$Hypertension + pheno_data3$Age + pheno_data3$Dyslipidemia + pheno_data3$Obesity
                + pheno_data3$CRD + pheno_data3$COPD, family = binomial)
  test.m <- lrtest( model1, model0 )
  statistic[k] <- tidy(model1)$statistic[2]
  plist[k] <- test.m$p.value
  betalist[k] <- coef(model1)[2] }

p_adjust <- p.adjust(as.matrix(plist), method ="fdr")

Diabetes_associat <- cbind(mz_rt,betalist, statistic, plist, p_adjust)

##### dyslipidemia ######

plist <- c(1:9893)
betalist <- c(1:9893)
statistic <- c(1:9893)

for (k in 1:9893) {
  model1 <- glm(pheno_data3$Dyslipidemia  ~  unlist(data_matrix3[k, 1:177]) + pheno_data3$Sex + pheno_data3$Heart_disease
                + pheno_data3$Hypertension + pheno_data3$Diabetes + pheno_data3$Age + pheno_data3$Obesity
                + pheno_data3$CRD + pheno_data3$COPD, family = binomial)
  model0 <- glm(pheno_data3$Dyslipidemia  ~   pheno_data3$Sex + pheno_data3$Heart_disease
                + pheno_data3$Hypertension + pheno_data3$Diabetes + pheno_data3$Age + pheno_data3$Obesity
                + pheno_data3$CRD + pheno_data3$COPD, family = binomial)
  test.m <- lrtest( model1, model0 )
  statistic[k] <- tidy(model1)$statistic[2]
  plist[k] <- test.m$p.value
  betalist[k] <- coef(model1)[2] }
```

```
p_adjust <- p.adjust(as.matrix(plist), method ="fdr")

Dyslipidemia_associat <- cbind(mz_rt,betalist, statistic, plist, p_adjust)

##### obesity ######

plist <- c(1:9893)
betalist <- c(1:9893)
statistic <- c(1:9893)

for (k in 1:9893) {
  model1 <- glm(pheno_data3$Obesity  ~  unlist(data_matrix3[k, 1:177]) + pheno_data3$Sex + pheno_data3$Heart_disease
               + pheno_data3$Hypertension + pheno_data3$Diabetes + pheno_data3$Dyslipidemia + pheno_data3$Age
               + pheno_data3$CRD + pheno_data3$COPD, family = binomial)
  model0 <- glm(pheno_data3$Obesity  ~   pheno_data3$Sex + pheno_data3$Heart_disease
               + pheno_data3$Hypertension + pheno_data3$Diabetes + pheno_data3$Dyslipidemia + pheno_data3$Age
               + pheno_data3$CRD + pheno_data3$COPD, family = binomial)
  test.m <- lrtest( model1, model0 )
  statistic[k] <- tidy(model1)$statistic[2]
  plist[k] <- test.m$p.value
  betalist[k] <- coef(model1)[2] }

p_adjust <- p.adjust(as.matrix(plist), method ="fdr")

Obesity_associat <- cbind(mz_rt,betalist, statistic, plist, p_adjust)


##### CRD ######

plist <- c(1:9893)
betalist <- c(1:9893)
statistic <- c(1:9893)

for (k in 1:9893) {
  model1 <- glm(pheno_data3$CRD  ~  unlist(data_matrix3[k, 1:177]) + pheno_data3$Sex + pheno_data3$Heart_disease
               + pheno_data3$Hypertension + pheno_data3$Diabetes + pheno_data3$Dyslipidemia + pheno_data3$Obesity
               + pheno_data3$Age + pheno_data3$COPD, family = binomial)
```

```
  model0 <- glm(pheno_data3$CRD  ~   pheno_data3$Sex + pheno_data3$Heart_disease
               + pheno_data3$Hypertension + pheno_data3$Diabetes + pheno_data3$Dyslipidemia + pheno_data3$Obesity
               + pheno_data3$Age + pheno_data3$COPD, family = binomial)
  test.m <- lrtest( model1, model0 )
  statistic[k] <- tidy(model1)$statistic[2]
  plist[k] <- test.m$p.value
  betalist[k] <- coef(model1)[2] }

p_adjust <- p.adjust(as.matrix(plist), method ="fdr")

CRD_associat <- cbind(mz_rt,betalist, statistic, plist, p_adjust)


##### COPD ######

plist <- c(1:9893)
betalist <- c(1:9893)
statistic <- c(1:9893)

for (k in 1:9893) {
  model1 <- glm(pheno_data3$COPD  ~  unlist(data_matrix3[k, 1:177]) + pheno_data3$Sex + pheno_data3$Heart_disease
               + pheno_data3$Hypertension + pheno_data3$Diabetes + pheno_data3$Dyslipidemia + pheno_data3$Obesity
               + pheno_data3$CRD + pheno_data3$Age, family = binomial)
  model0 <- glm(pheno_data3$COPD  ~   pheno_data3$Sex + pheno_data3$Heart_disease
               + pheno_data3$Hypertension + pheno_data3$Diabetes + pheno_data3$Dyslipidemia + pheno_data3$Obesity
               + pheno_data3$CRD + pheno_data3$Age, family = binomial)
  test.m <- lrtest( model1, model0 )
  statistic[k] <- tidy(model1)$statistic[2]
  plist[k] <- test.m$p.value
  betalist[k] <- coef(model1)[2] }

p_adjust <- p.adjust(as.matrix(plist), method ="fdr")

COPD_associat <- cbind(mz_rt,betalist, statistic, plist, p_adjust)

#### compute metabolite features affected by confounding variables###
library(epicalc)
```

```
library(broom)

data_matrix3 <- data_matrix[,-129:-148]
pheno_data3 <- pheno_data[-129:-148,]

###Age####
betalist_crude <- c(1:9893)
betalist_adj <- c(1:9893)
for (k in 1:9893) {
  model <- glm(pheno_data3$Infection_status_code  ~  unlist(data_matrix3[k, 1:177]), family = binomial)
  model1<- glm(pheno_data3$Infection_status_code  ~  unlist(data_matrix3[k, 1:177])+ pheno_data3$Age, family = binomial)
  betalist_crude[k] <- coef(model)[2]
  betalist_adj[k] <- coef(model1)[2] }
Age_confounding <- cbind(betalist_crude, betalist_adj)
colnames(Age_confounding) <- c("Crude_effect", "Adjusted_effect")
percentage_difference_age <- ((Age_confounding[,1]-Age_confounding[,2])/Age_confounding[,1])*100


###Sex####
betalist_crude <- c(1:9893)
betalist_adj <- c(1:9893)
for (k in 1:9893) {
  model <- glm(pheno_data3$Infection_status_code  ~  unlist(data_matrix3[k, 1:177]), family = binomial)
  model1<- glm(pheno_data3$Infection_status_code  ~  unlist(data_matrix3[k, 1:177])+ pheno_data3$Sex, family = binomial)
  betalist_crude[k] <- coef(model)[2]
  betalist_adj[k] <- coef(model1)[2] }
Sex_confounding <- cbind(betalist_crude, betalist_adj)
colnames(Sex_confounding) <- c("Crude_effect", "Adjusted_effect")
percentage_difference_sex <- ((Sex_confounding[,1]-Sex_confounding[,2])/Sex_confounding[,1])*100

###Heart_disease####
betalist_crude <- c(1:9893)
betalist_adj <- c(1:9893)
for (k in 1:9893) {
  model <- glm(pheno_data3$Infection_status_code  ~  unlist(data_matrix3[k, 1:177]), family = binomial)
  model1<- glm(pheno_data3$Infection_status_code  ~  unlist(data_matrix3[k, 1:177])+ pheno_data3$Heart_disease, family = binomial)
  betalist_crude[k] <- coef(model)[2]
```

```
  betalist_adj[k] <- coef(model1)[2] }
Heart_disease_confounding <- cbind(betalist_crude, betalist_adj)
colnames(Heart_disease_confounding) <- c("Crude_effect", "Adjusted_effect")
percentage_difference_Heart_disease <- ((Heart_disease_confounding[,1]-Heart_disease_confounding[,2])/Heart_disease_confounding[,1])*100

###Hypertension####
betalist_crude <- c(1:9893)
betalist_adj <- c(1:9893)
for (k in 1:9893) {
  model <- glm(pheno_data3$Infection_status_code  ~  unlist(data_matrix3[k, 1:177]), family = binomial)
  model1<- glm(pheno_data3$Infection_status_code  ~  unlist(data_matrix3[k, 1:177])+ pheno_data3$Hypertension, family = binomial)
  betalist_crude[k] <- coef(model)[2]
  betalist_adj[k] <- coef(model1)[2] }
Hypertension_confounding <- cbind(betalist_crude, betalist_adj)
colnames(Hypertension_confounding) <- c("Crude_effect", "Adjusted_effect")
percentage_difference_Hypertension <- ((Hypertension_confounding[,1]-Hypertension_confounding[,2])/Hypertension_confounding[,1])*100

###Diabetes####
betalist_crude <- c(1:9893)
betalist_adj <- c(1:9893)
for (k in 1:9893) {
  model <- glm(pheno_data3$Infection_status_code  ~  unlist(data_matrix3[k, 1:177]), family = binomial)
  model1<- glm(pheno_data3$Infection_status_code  ~  unlist(data_matrix3[k, 1:177])+ pheno_data3$Diabetes, family = binomial)
  betalist_crude[k] <- coef(model)[2]
  betalist_adj[k] <- coef(model1)[2] }
Diabetes_confounding <- cbind(betalist_crude, betalist_adj)
colnames(Diabetes_confounding) <- c("Crude_effect", "Adjusted_effect")
percentage_difference_Diabetes <- ((Diabetes_confounding[,1]-Diabetes_confounding[,2])/Diabetes_confounding[,1])*100

###Dyslipidemia####
betalist_crude <- c(1:9893)
betalist_adj <- c(1:9893)
for (k in 1:9893) {
  model <- glm(pheno_data3$Infection_status_code  ~  unlist(data_matrix3[k, 1:177]), family = binomial)
  model1<- glm(pheno_data3$Infection_status_code  ~  unlist(data_matrix3[k, 1:177])+ pheno_data3$Dyslipidemia, family = binomial)
  betalist_crude[k] <- coef(model)[2]
  betalist_adj[k] <- coef(model1)[2] }
```

```
Dyslipidemia_confounding <- cbind(betalist_crude, betalist_adj)
colnames(Dyslipidemia_confounding) <- c("Crude_effect", "Adjusted_effect")
percentage_difference_Dyslipidemia <- ((Dyslipidemia_confounding[,1]-Dyslipidemia_confounding[,2])/Dyslipidemia_confounding[,1])*100

###Obesity####
betalist_crude <- c(1:9893)
betalist_adj <- c(1:9893)
for (k in 1:9893) {
  model <- glm(pheno_data3$Infection_status_code  ~  unlist(data_matrix3[k, 1:177]), family = binomial)
  model1<- glm(pheno_data3$Infection_status_code  ~  unlist(data_matrix3[k, 1:177])+ pheno_data3$Obesity, family = binomial)
  betalist_crude[k] <- coef(model)[2]
  betalist_adj[k] <- coef(model1)[2] }
Obesity_confounding <- cbind(betalist_crude, betalist_adj)
colnames(Obesity_confounding) <- c("Crude_effect", "Adjusted_effect")
percentage_difference_Obesity <- ((Obesity_confounding[,1]-Obesity_confounding[,2])/Obesity_confounding[,1])*100

###COPD####
betalist_crude <- c(1:9893)
betalist_adj <- c(1:9893)
for (k in 1:9893) {
  model <- glm(pheno_data3$Infection_status_code  ~  unlist(data_matrix3[k, 1:177]), family = binomial)
  model1<- glm(pheno_data3$Infection_status_code  ~  unlist(data_matrix3[k, 1:177])+ pheno_data3$COPD, family = binomial)
  betalist_crude[k] <- coef(model)[2]
  betalist_adj[k] <- coef(model1)[2] }
COPD_confounding <- cbind(betalist_crude, betalist_adj)
colnames(COPD_confounding) <- c("Crude_effect", "Adjusted_effect")
percentage_difference_COPD <- ((COPD_confounding[,1]-COPD_confounding[,2])/COPD_confounding[,1])*100

###CRD####
betalist_crude <- c(1:9893)
betalist_adj <- c(1:9893)
for (k in 1:9893) {
  model <- glm(pheno_data3$Infection_status_code  ~  unlist(data_matrix3[k, 1:177]), family = binomial)
  model1<- glm(pheno_data3$Infection_status_code  ~  unlist(data_matrix3[k, 1:177])+ pheno_data3$CRD, family = binomial)
  betalist_crude[k] <- coef(model)[2]
  betalist_adj[k] <- coef(model1)[2] }
CRD_confounding <- cbind(betalist_crude, betalist_adj)
```

```
colnames(CRD_confounding) <- c("Crude_effect", "Adjusted_effect")
percentage_difference_CRD <- ((CRD_confounding[,1]-CRD_confounding[,2])/CRD_confounding[,1])*100
```
